# Ensemble machine learning methods in screening electronic health records: A scoping review

**DOI:** 10.1177/20552076231173225

**Published:** 2023-05-09

**Authors:** Christophe AT Stevens, Alexander RM Lyons, Kanika I Dharmayat, Alireza Mahani, Kausik K Ray, Antonio J Vallejo-Vaz, Mansour TA Sharabiani

**Affiliations:** 1 Imperial Centre for Cardiovascular Disease Prevention (ICCP), Department of Primary Care and Public Health, School of Public Health, 4615 Imperial College London, London, UK; 2 Quantitative Research, Davidson Kempner Capital Management, New York, NY, USA; 3 Department of Medicine, Faculty of Medicine, 16778 University of Seville, Sevilla, Spain; 4 Clinical Epidemiology and Vascular Risk, Instituto de Biomedicina de Sevilla (IBiS), IBiS/Hospital Universitario Virgen del Rocío/Universidad de Sevilla/CSIC, Sevilla, Spain; 5 Department of Primary Care and Public Health, School of Public Health, 4615 Imperial College London, London, UK

**Keywords:** Ensemble machine learning, supervised machine learning, mass screening, electronic health records, scoping review

## Abstract

**Background:**

Electronic health records provide the opportunity to identify undiagnosed individuals likely to have a given disease using machine learning techniques, and who could then benefit from more medical screening and case finding, reducing the number needed to screen with convenience and healthcare cost savings. Ensemble machine learning models combining multiple prediction estimates into one are often said to provide better predictive performances than non-ensemble models. Yet, to our knowledge, no literature review summarises the use and performances of different types of ensemble machine learning models in the context of medical pre-screening.

**Method:**

We aimed to conduct a scoping review of the literature reporting the derivation of ensemble machine learning models for screening of electronic health records. We searched EMBASE and MEDLINE databases across all years applying a formal search strategy using terms related to medical screening, electronic health records and machine learning. Data were collected, analysed, and reported in accordance with the PRISMA scoping review guideline.

**Results:**

A total of 3355 articles were retrieved, of which 145 articles met our inclusion criteria and were included in this study. Ensemble machine learning models were increasingly employed across several medical specialties and often outperformed non-ensemble approaches. Ensemble machine learning models with complex combination strategies and heterogeneous classifiers often outperformed other types of ensemble machine learning models but were also less used. Ensemble machine learning models methodologies, processing steps and data sources were often not clearly described.

**Conclusions:**

Our work highlights the importance of deriving and comparing the performances of different types of ensemble machine learning models when screening electronic health records and underscores the need for more comprehensive reporting of machine learning methodologies employed in clinical research.

## Introduction

Electronic health records (EHRs), unlike paper-based systems, are not only convenient for storing and managing patient records^1,2^ but also allow the application of machine learning (ML) techniques to pre-screen undiagnosed individuals more likely to have a given disease based on their available demographic, clinical and lifestyle variables. ML techniques have often shown high predictive performance and allow the management of a large number of variables in an automated fashion,^3–7^ and could identify individuals more likely to benefit from a referral to their medical specialist for clinical screening or diagnostic confirmation.

All ML models are derived using algorithms applied to samples from populations with given diseases plus controls. However, unlike traditional ML algorithms that generate one model, ensemble ML algorithms generate multiple models called ‘weak’ or ‘base’ learners^8^ and combine their predictions into one single value. Predictions originating from ensemble ML models (EMLs) are expected to retain good predictive performances when applied to samples drawn from similar populations and are thus said to have better generalisation performances.^9^ An analogy of EML can be found in panels of a medical expert, whose diagnoses are expected to be less erroneous (i.e. predict a disease/outcome with greater sensitivity and specificity) on average than if a single expert were to be consulted. EMLs's potential enhanced predictive and generalisation performances rely not only on the choice of an optimal ML algorithm but also on the selection of a dataset representative of the population and on the pre-processing steps (Box 1).^10–14^

Box 1. Pre-processing steps to derive machine learning (ML) models.

- **Choice of Software:** different ML algorithms are being implemented in one or more statistical software environments (e.g. R, Python and Matlab).
- **Handling of missing data:** given some conditions, the missing values of variables in a dataset can be replaced by new values. Median replacement can be viewed as one of the simplest and perhaps less accurate examples.
- **Feature engineering:** Creating a new predictor variable based on existing variables, for instance, using domain knowledge. Computing body mass index based on weight and height can be seen as one of the simplest examples of feature engineering.
- **Data preparation**^a^**:** cleaning and transforming data to make it more suitable to be fed to an algorithm. For example, scaling continuous variables between 0 and 1, before feeding them to a neural network.
- **Handling of class-imbalance:** when the cases and controls are highly disproportionate (e.g. 1% vs 99% respectively) the data is class-imbalanced which can negatively impact the training of the model. There are different ways of handling this problem. One of the simplest could be replicating instances of the minority class to overrepresent them.
- **Data partitioning:** division of the data into multiple non-overlapping sets in order to derive, tune and evaluate model performances on different datasets.
- **Choice of metrics:** finding suitable metrics that appropriately and fairly evaluate the performances of ML models. For example, use of the Area Under the Receiver Operating Characteristic Curve to evaluate the performance of a binary classifier.
- **Feature selection:** finding and selecting the variables without spurious association with the response variable to improve model performances.
- **Hyperparameters tuning:** finding and selecting an optimal value for a hyperparameter of an ML algorithm.

^a^The data preparation step here excludes feature engineering, handling of missing data and feature selection which are described in other steps.

EMLs are highly complex and their structures and compositions are difficult to be described consistently^15^; therefore it seems reasonable to use the taxonomy proposed by Kuncheva^16^ to classify ensembles according to four clearly defined ‘levels’ or dimensions (combination, classifier, data and feature) (Box 2).

Box 2. Taxonomy of ensembles in four dimensions.

**Combination:** EMLs can either be composed of models arranged sequentially or in parallel:

- *Sequentially (aka boosting):* Boosting aims to boost prediction performance by using the output probabilities of its models (called weak learners) as an input for the subsequent weak learners in the sequence, thus, practically improving on their predecessor(s) mistake(s).
- *Parallel:* Models arranged in parallel are called ‘base learners’ and their predictions could be fused into one using different fusion strategies^17^: (i) majority voting when each model cast a vote, the ensemble returns the outcome with the greatest number of votes, (ii) average probability when the ensemble average over the probabilities, rather than votes, returned by each base learner, (iii) weighted probability when the base learner probabilities are weighted using a chosen performance metric, and (iv) stacking when a new model called meta-learner is used to combine the predictions of base learners.

**Classifier:** Models in an ensemble can either all be derived using the same or different ML algorithms (i.e. homogeneous vs. heterogeneous classifiers, respectively). It is worth noting that classifiers used in sequential ‘boosting’ ensembles typically are homogeneous.

**Data:** Data in this context refer to the sampling strategy used to train the base learners’ models. Base learners of parallel ensembles can either all be trained on the same or different datasets using a popular method, named bootstrap aggregation or ‘bagging’,^18,19^ which consists in deriving each base learner with bootstrapped samples.

**Feature:** It is possible to derive each base learner in a parallel ensemble using different features or variables, either randomly or programmatically. A famous ML algorithm, called random forest (RF),^20,21^ randomly selects features to be used within each of its base learners. It is worth noting that RF also use bagging.

Although EMLs are often reported to outperform other ML models, the literature on the use of EML methods for pre-screening diseases within EHRs is limited, and previous reviews have instead focused on the application of any ML models,^22–24^ or specifically on deep ML models to identify diseases within medical data,^25^ and EHRs.^26^ Among the few available reviews partly summarising the use of EMLs, Yang et al., found that there was a 19% increase in EMLs models reported between 2009–2014 versus 2015–2019,^22^ Hossain et al., stated that the random Forest EML algorithm ‘is one of the most accurate ML-based algorithms’,^23^ and Nwanosike et al., found that the XGBoost and RandomForest EML algorithms showed the highest potential for clinical application.^24^

The enhanced predictive performances of EMLs come with a computational cost. However, with the increased trend in computational power and availability of big data in the form of EHRs, an increasing trend in the application of EMLs is expected. Gaining new insights into the differences in predictive performances between EMLs and with non-ensemble ML models could potentially help predictive screening researchers design EMLs capable of identifying diseases in EHRs with greater predictive performance. This systematic scoping review, therefore, summarises the very specific field of EMLs applied to the pre-screening of EHRs to identify undiagnosed individuals, and aims to inform future research with four main objectives: (i) to assess the extent, nature, and performances of different types of EMLs; (ii) to provide a complete picture of what pre-processing steps have been applied prior to deriving, testing, selecting, and comparing ML models; (iii) to characterise the data sources used to derive EMLs; and (iv) to assess whether the Transparent Reporting of a multivariable prediction model for Individual Prognosis Or Diagnosis (TRIPOD) statement^27^ is used to report EMLs.

## Methods

### Literature search

A literature search was conducted in the databases MEDLINE and EMBASE for full-text original research peer-reviewed articles describing the development or validation of an EML for medical pre-screening of potential undiagnosed patients in EHRs. Articles concerned with disease prognosis (i.e. time to event), disease sub-classification, treatment allocation or that were not specific to a given disease (e.g. all-cause mortality, drug-induced disease, a disease caused by human intervention) were excluded. Simulation studies that did not derive an ML model from a real-world dataset were also excluded. The following search strategy was run using OvidSP including multipurpose keywords: #1 (‘machine learning’ or ‘boosting’ or ‘ensemble’ or ‘bagging’ or ‘stacking’ or ‘random forest’ or ‘super learner’ or ‘base learner’ or ‘meta learner’ or ‘weak learner’).mp. #2 (screening* or identif* or find*).mp. #3 (‘health record’ or ‘medical record’ or ‘patient record’ or ‘GP record’ or ‘primary care’).mp. #4 (1 & 2 & 3). Databases were searched from their inception to 11 April 2022. No limits were included in terms of language. This scoping review followed an unpublished and unregistered protocol.

### Literature selection

**Figure 1.**
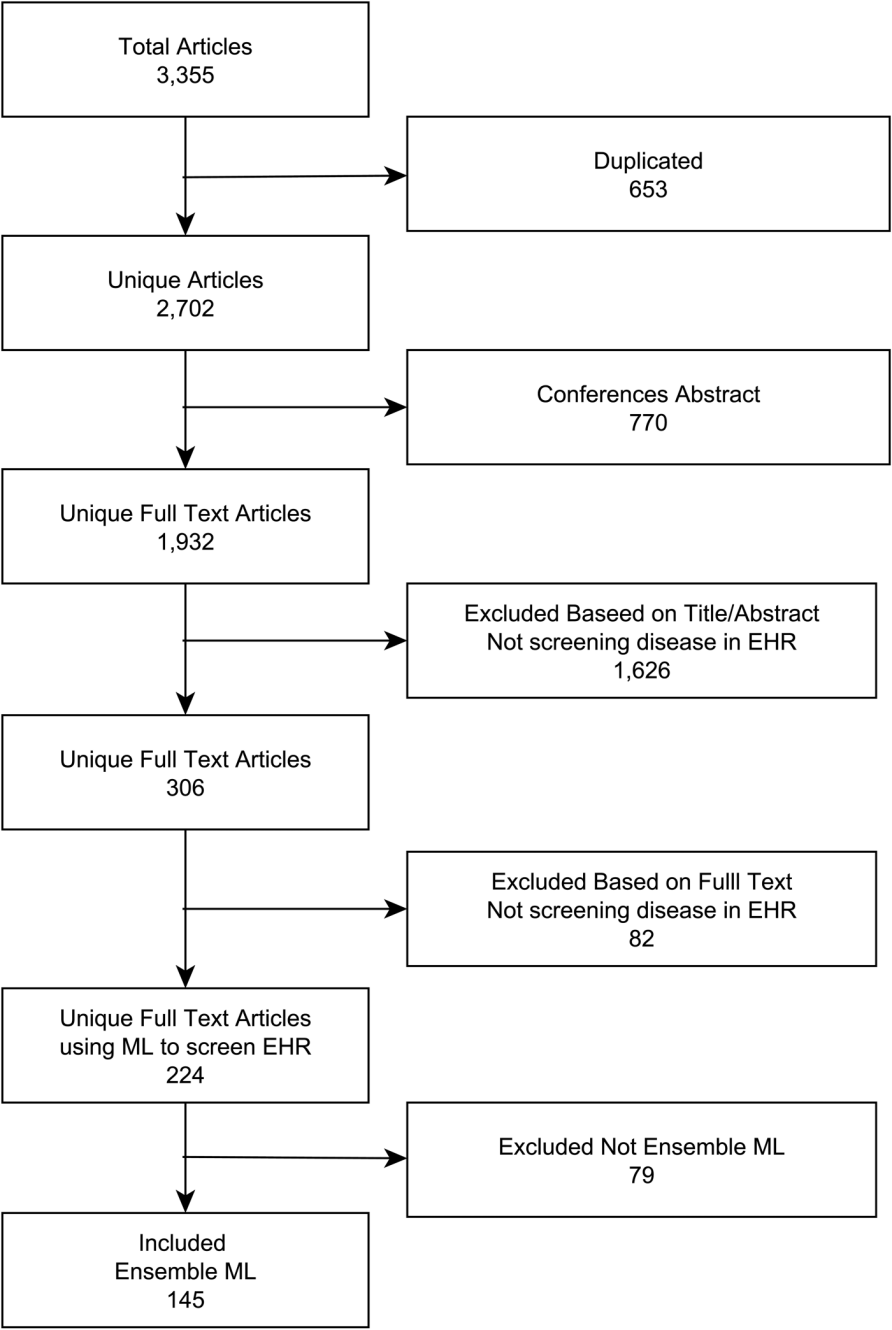
Flowchart of articles inclusion and exclusion.

A single reviewer performed the article selection using the EndNote software. From the articles retrieved, duplicated articles and conference abstracts were discarded in the first step (Figure 1). The remaining articles were then subjected to screening based on titles and abstracts and they were excluded if they did not match the inclusion criteria or had any exclusion criteria; however, at this stage articles using ML methods were retained for inclusion regardless of whether the model was an ensemble. The articles that progressed from the prior step were then subjected to full-text review. In the final step, the articles were separated into two groups: (i) articles that reported non-EML only, and (ii) articles that reported at least one EML (Figure 1). By proceeding in this way, it was possible to evaluate the proportion of ML articles describing at least one EML model.

### Data charting

The data from the articles reporting on at least one EML were extracted by a single researcher into a Microsoft Access database through a bespoke data collection form. The access database is available in the Mendeley Academic Repository^28^ and its diagram is available in Supplemental Figure 3.

### Data synthesis

Due to the considerable variety in the methods used for the derivation of EMLs and due to the exploratory nature of scoping reviews, it was not always possible to define categories for each field in advance. The disease classification scheme can be found in Supplemental Table 2. The data extractor added every article characteristic as found in their full text and defined the categories a posteriori to facilitate the analysis. The classification of ensemble methods has been determined a priori, using the taxonomy proposed by Kuncheva.^16^

### Data analysis

The content of the final database was analysed and presented as tables and figures using the R language and environment for Statistical Computing^29^ (version 3.6.0). The cumulative hypergeometric test has been used to establish whether a specific algorithm was selected as the best model in as many publications as observed due to chance alone.^30^

### Data reporting and availability

This article followed the PRISMA for Scoping Reviews guideline^31^ which checklist can be found in Supplemental Checklist 1.

The data that support the findings of this study are openly available in the Mendeley Academic Repository at http://doi.org/10.17632/ny6nmjwhdx.1.

## Results

### Article selection

After applying a formal search strategy (described in detail in the methods section), a total of 3355 articles were retrieved (Figure 1), of which 653 were duplicated, 770 were conference abstracts, and 1626 did not meet the inclusion criteria based on titles and abstracts. The texts of the 306 remaining articles were read fully, of which a further 82 did not meet the inclusion criteria. This led to 224 articles that described the use of ML models for medical pre-screening in EHRs. Of them, 145 described the development of at least one EML (64.7%) and were finally subjected to data extraction and included in the analyses (Supplemental References 1 & 2).

### Use of ensembles

**Figure 2.**
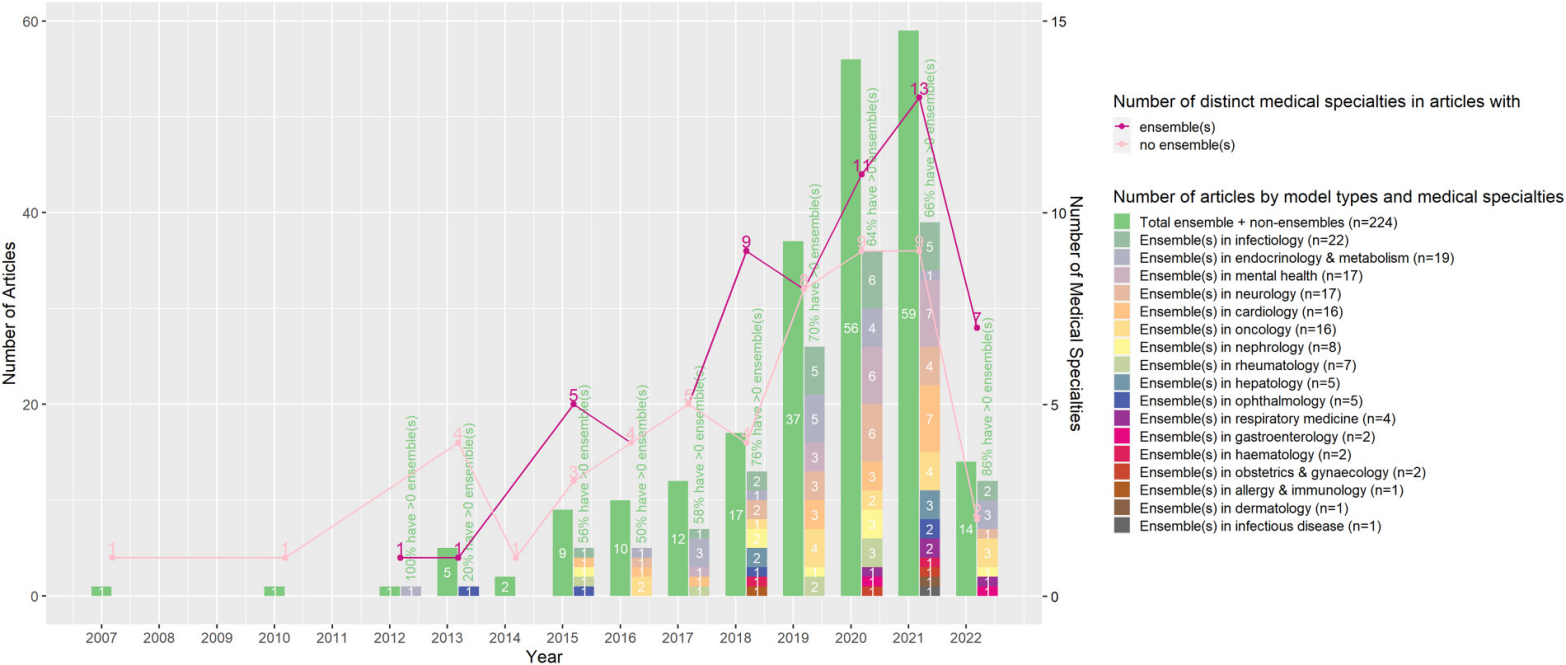
Use of ensemble and non-ensemble ML models in EHRs over time by medical specialty.

ML: machine learning; EHRs: electronic health records.

Despite searching across all publication years the first publication reporting on an EML was in 2012 in the field of endocrinology and metabolism. Since then, our findings show that published EML usage in EHRs has increased progressively, particularly in the field of infectious disease, endocrinology and metabolism, mental health, neurology, cardiology, and oncology (Figure 2). The emerging trend in the use of EML seems to follow that of ML models in general, yet EMLs are used across more medical specialties. Articles predominantly reported on studies where EML models were both developed and validated (n = 105, 72.4%), albeit mostly with internal validation (n = 79, 54.5%) rather than external validation (n = 20, 13.8%) (Supplemental Table 1). 26.9% (n = 39) of the articles described the development of EMLs without validation.

### Type and performances of EML

Among the 145 included articles, 108 (74.5%) compared different models. Of them, 67 (62.0%) reported that an EML model outperformed a non-ensemble model.

### Dimension of ensembles

#### Combination

Table 1 shows the number of articles reporting on at least one EML in each dimension. More articles were reported on parallel ensembles than on sequential ensembles (n = 118, 81.4% vs. n = 81, 55.9%, respectively). Among articles comparing performances across models, sequential EMLs were more likely to be selected as best models (subjectively by studies’ authors and for their intended purpose) than parallel EMLs (46.3% vs. 39.8%, respectively), and the quality of their analyses, in terms of data partitioning strategies (Box 1), was better (quality is 75.8% for sequential EMLs, vs. 64.9% for parallel EMLs). Most parallel EMLs used the majority voting technique as a fusion strategy (73.8%); averaged and weighted probabilities fusion and stacking were used to a much lower extent (4.8%, 4.1% and 4.1%, respectively). The chances of being chosen as the best model were much higher for EML using weighted probabilities fusion (100.0%) and stacking (75.0%) compared to other ML models including sequential EMLs. In terms of data partitioning strategy, the quality of the analyses was higher in studies that combined parallel base learners using the averaged probability fusion (83.5%) compared to studies using weighted vote (60%), majority voting (66.0%) and stacking (66.7%).

**Table 1.** The number of articles that used, compared, and had an ensemble machine learning model (EML) as their best model by dimension.

		Total articles		Articles reporting comparison of EML against other models		Articles reporting that an EML was best	
		*N* = 145					
Dimension	Dimension Type	*N* articles (%)	Quality^a^ (%)	N articles (%)	Quality^a^ (%)	N articles (%)	Quality^a^ (%)
Combination	Sequential (boosting)	81 (55.9)	72.2	67 (82.7)	75.4	31 (46.3)	75.8
	Parallel	118 (81.4)	63.1	93 (78.8)	68.3	37 (39.8)	64.9
	→Parallel Maj Vote	107 (73.8)	64.5	89 (83.2)	70.2	25 (28.1)	66.0
	→Parallel Average Probability	7 (4.8)	64.3	3 (42.9)	83.3	3 (100.0)	83.3
	→Parallel Weighted Vote	6 (4.1)	58.3	5 (83.3)	60.0	5 (100.0)	60.0
	→Parallel Stacking	6 (4.1)	83.3	4 (66.7)	75.0	3 (75.0)	66.7
Classifier	Heterogeneous classifiers (base learners)	14 (9.7)	82.1	11 (78.6)	81.8	9 (81.8)	77.8
	Homogeneous classifiers (base learners)	142 (97.9)	64.1	108 (76.1)	69.9	59 (54.6)	68.6
Data	Data sampling (bagging)	112 (77.2)	62.9	89 (79.5)	68.5	27 (30.3)	59.3
	No data sampling (no bagging)	89 (61.4)	72.5	73 (82.0)	75.3	41 (56.2)	76.8
Feature	Random feature selection	108 (74.5)	63.9	89 (82.4)	69.1	26 (29.2)	61.5
	No random feature selection	93 (64.1)	71.5	74 (79.6)	75.0	42 (56.8)	75.0

^a^ Quality is the sum of single points attributed to an article for (1) describing a suitable data partitioning strategy in training and evaluating their model and for (2) describing tuning of hyperparameters. The highest quality of a publication is 2 and the lowest is 0, here the average score is shown in percent.

#### Classifier

EMLs were mostly homogeneous in their classifiers (97.9% of articles, vs. heterogeneous: 9.7%). However, when compared, heterogeneous EMLs had higher chances of being the best models for the intended purposes than homogeneous ensembles (81.8% vs. 54.6%, respectively) and these articles had a more systematic and robust methodology in terms of data partitioning to prevent against overfitting (quality was 77.8% vs. 68.6%, respectively) (Table 1).

#### Data and random feature

As shown in Table 1, counts and proportions of bagging and random feature are similar as most of these studies use the random forest classifier which implements both bagging and random feature selection. No advantages were observed for the use of bagging and random feature selection, with low proportions being the best model for the intended purposes (30.3% for bagging vs. 56.2%for non-bagging, and 29.2% for random feature selection vs. 56.8% when not using random feature selection). Articles that described bagging and random feature selection overall had a lower quality in terms of methodology to prevent against overfitting (59.3% vs. 76.8% for bagging vs. non-bagging; 61.5% for random feature selection vs. 75% for not using random feature selection).

### EML algorithms

Table 2 shows the proportions of articles reviewed where each ML algorithm was used to derive a base/weak/meta-learner by type of ensemble. Boosting/sequential ensembles were exclusively made of decision trees weak learners (100%) and do not have meta-learners. Heterogeneous ensembles were mostly made of random forest (28.2%), logistic regression (25.6%), artificial neural network (20.5%) and, to a lesser extent, of gradient boosting and naïve Bayes models (12.8% each). In heterogeneous EMLs, when a meta-learner was used (i.e. stacking), its algorithm was gradient boosting (25%), XGBoost (25%), and not specified in 50% of articles. Ensembles made with bagging and random feature were mostly derived using the random forest algorithm and thus decision trees were predominantly used as base learners (92.1% of articles in bagging, 99.1% of articles in random features). However, some articles reporting a bagging EML also used neural networks (2.7%), LASSO penalised regression, random forest, and XGBoost (1.8% each) as base learners. One article reported bagging and another reported that a random feature EML used XGBoost as a meta-learner.

**Table 2.** The number of articles where each Machine Learning (ML) algorithm was used for the derivation of base/weak/meta learners by type of ensemble.

Ensemble type	Learner level	Algorithm used	*N* articles in dimension (%)
Boosting	Weak learner	Decision tree	91 (100.0)
	Meta learner	None	0 (0.0)
Heterogeneous classifier	Base learner	Random forest	11 (28.2)
		Logistic regression	10 (25.6)
		Artificial neural network	8 (20.5)
		Gradient boosting	5 (12.8)
		Naïve Bayes	5 (12.8)
	Meta learner	Not given	2 (50.0)
		Gradient boosting	1 (25.0)
		XGBoost	1 (25.0)
Bagging	Base learner	Decision tree	105 (92.1)
		Artificial neural network	3 (2.7)
		LASSO	2 (1.8)
		Random forest	2 (1.8)
		XGBoost	2 (1.8)
	Meta learner	XGBoost	1 (100.0)
Random features	Base learner	Decision tree	107 (99.1)
		Artificial neural network	1 (0.9)
	Meta learner	XGBoost	1 (100.0)

Table 3 displays the value of the cumulative hypergeometric test, which establishes the probability that an algorithm is selected as the best model for the intended purpose. The performances of the custom parallel ensemble with a weighted average fusion of base learners and XGBoost performed better than by random chances (*p*-value <0.001 for both comparisons). Average probability fusion (*p*-value: 0.003), gradient boosting (*p*-value: 0.02), deep learning (*p*-value: 0.02), other custom/non-ensemble algorithms (*p*-value: 0.02), and stacking (*p*-value: 0.07) did all perform better than random chances.

**Table 3.** Hypergeometric test to establish the likelihood for each algorithm to be selected as the best model as many times or more times than they were actually selected. A low *p*-value indicates that an algorithm has a very low chance to be selected as many times as observed by chance alone.

A	K	M	N	x	Two-sided test
Algorithms compared in the studies included in the scoping review	Number of articles where this algorithm A was compared and could have been chosen as the best model	Number of models generated by the algorithm A and compared to N models generated by other algorithms in K	Number of models generated by other algorithms and compared to the M models generated by this algorithm in K articles	Number of articles in K where a model generated by the algorithm A was selected as the best model	*P*(*X*> = *x*) × 2, that is, the probability that the number of models generated by algorithm A were observed as best models by chance
Fusion: weighted vote	5	5	22	5	<0.001
XGBoost	37	37	128	18	<0.001
Fusion: average probability	3	3	14	3	0.003
Gradient boosting	28	28	106	11	0.02
Deep learning	15	15	58	7	0.02
Other	4	4	17	3	0.02
Stacking	4	5	14	3	0.07
Random forest	90	91	335	25	0.13
Elastic net	8	8	35	3	0.31
LASSO	17	17	67	5	0.46
CatBoost	1	1	3	1	0.5
Rule based	5	5	14	2	0.79
Random tree	2	2	7	1	0.83
Artificial neural network	27	27	106	6	0.97
Multilayer perceptron	14	14	59	3	1
Ridge regression	6	6	34	1	1
Naïve bayes	30	30	141	2	1
Support vector machine	44	44	198	3	1
Logistic regression	66	66	245	6	**1**

### Pre-processing steps

#### Hyperparameter tuning

In around half the selected articles authors reported tuning the hyperparameters of their ML models (n = 76; 52.4%) or tuning the hyperparameters for some of their models only (n = 6, 4.1%) (Table 4). In one-third of the articles, hyperparameter tuning was not described (n = 42, 29.0%). When tuning was not used (n = 21, 14.5%), hyperparameters were set to their default values (n = 14; 9.7%), or an arbitrary value was chosen (n = 5; 3.4%).

**Table 4.** Methods applied in the pre-processing steps.

Pre-processing step	Methods used? (only top five methods in table)	*N* articles (%)	Pre-processing step	Methods used? (only top five methods in table)	*N* articles (%)	Pre-processing step	Methods used? (only top five methods in table)	*N* articles (%)
Choice of software	Not reported	33 (22.8)	Data Partitioning strategy	CV + Held-out dataset	59 (40.7)	Hyper-parameter tuning	Used	76 (52.4)
	Yes, was Reported	112 (77.2)		Held out only	39 (26.9)		Not described	42 (29.0)
	➔Python	65 (44.8)		CV only	34 (23.4)		Not used	21 (14.5)
	➔R	35 (24.1)		Not clear	13 (9.0)		➔Default values	14 (9.7)
	➔WEKA	7 (4.8)					➔Arbitrary values	5 (3.4)
	➔Matlab	2 (1.4)					➔No details	2 (1.4)
	➔DaVinci Labs	1 (0.7)					Partly used	6 (4.1)
Handling of Missing Data^a^	Yes, was used	61 (42.1)	Data Partitioning Strategy: Cross-Validation^a^	Used	93 (64.1)	Hyperparameter tuning used: Search Type	Described	53 (36.6)
	➔Exclusion of records	24 (16.6)		➔10-fold	36 (24.8)		➔Grid search	46(31.7)
	➔Mean imputation	16 (11.0)		➔5-folds	33 (22.8)		➔Bayesian optim.	4 (2.8)
	➔Median imputation	8 (5.5)		➔Not detailed	18 (12.4)		➔Random Search	2 (1.4)
	➔K-nearest neighbours	7 (4.8)		➔3-fold	2 (1.4)		➔Manual search	1 (0.7)
	➔Handled by classifier	7 (4.8)		➔4-fold	2 (1.4)		Not described	92 (63.4)
	➔Multiple imputation by chained equations (MICE)	4 (2.8)		➔Multiple	1 (1.1)			
	Not described	60 (41.4)		➔Leave-one-out	1 (1.1)			
	Not used	24 (16.6)		Not used	39 (26.9)			
	➔Handled by classifier	9 (6.2)		➔Not alternative given	32 (22.1)			
	➔Non-applicable (e.g. text)	8 (5.5)		➔Bootstrapping	7 (4.8)			
	➔No missing	4 (2.8)		Not clear/described	13 (9.0)			
	➔No further detailed	3 (2.1)						
Data Preparation^a^	Not described	79 (54.5)	Choice of metrics^a^	Reported	125 (100)	Hyperparameter tuning: Cross-validation	Not Described	87 (60.0)
	Described	66 (45.5)		➔AUC	116 (80.0)		Used	56 (38.6)
	➔Scaling (e.g. normalisation)	24 (16.6)		➔Sensitivity	86 (59.3)		Not used	2 (1.4)
	➔One-hot encoding/dummy	19 (13.1)		➔PPV	66 (45.5)			
	➔Text-specific	17 (11.7)		➔Specificity	59 (40.7)			
	➔Discretisation	6 (4.1)		➔Accuracy	41 (28.3)			
	➔Time-series-specific	3 (2.1)		Not reported	0 (0)			
Handling of class imbalance^a^	Not given/described	73 (50.3)	Automated Feature Selection^a^	Not used/described	75 (51.7)			
	Yes, used/described	68 (46.9)		Yes, Used^a^	70 (48.3)			
	➔Down-sampling	16 (11.0)		➔Stepwise selection	15 (10.3)			
	➔SMOTE	16 (11.0)		➔LASSO	10 (6.9)			
	➔Matched instances	15 (10.3)		➔Information gain	9 (6.2)			
	➔Up-sampling	9 (6.2)		➔Handled by classifier	9 (6.2)			
	➔Random sampling	7 (4.8)		➔Random Forest	8 (5.5)			
	No imbalance	4 (2.8)						
	

^a^ Percentages in this category can necessarily be added up as each article can report more than one method.

The hyperparameter search strategy was not described in 19 articles (30.6%), grid search was reported in 46 articles (31.7%), and Bayesian optimisation, random and manual search were used in 4 (2.8%), 2 (1.4%) and 1 (0.7%) articles, respectively. Cross-validation used to find the best hyperparameters’ s values were reported in 56 articles (38.6%), not used in two articles (1.4%), and not described in 87 articles (60.0%).

#### Feature selection

Automated feature selection was not described or described as being manually performed in 75 articles (51.7%) (Table 4). The five most common feature selection methods were stepwise selection, LASSO penalised regression, stepwise selection using information gain, feature selection handled by a classifier, and random forest.

#### Choice of metrics

Metrics used to assess models’ performances were varied and numerous but four seem to be preferred by authors (Table 4): Area Under the Receiver Operating Characteristics Curve (AUC ROC), Sensitivity, Positive Predictive Values, and Specificity. The ROC curve and precision–recall curve were also commonly used, but we did not count their use as they are not metrics.

#### Data partitioning

Table 4 summarises the data partitioning strategies used in articles. 40.7% (n = 59) of articles reported the joint use of cross-validation and held-out dataset(s). 26.9% (n = 39) of articles reported the use of held-out dataset(s) in addition to their training and validation datasets without using cross-validation. 23.4% of articles (n = 34) used cross-validation only without held-out datasets. In 10 articles, the data partitioning strategies used were not clear (n = 13; 9%). When cross-validation was not used, seven articles (4.8%) reported using bootstrapping whereas it was not clear what method was used in 32 articles (22.1%). Most articles that reported the use of cross-validation either used 10-fold (n = 36; 24.8%), 5-fold (n = 33; 22.8%), or did not further describe it (n = 18; 12.4%).

#### Handling class imbalance

Methods used to deal with class imbalance were not described in 50.3% of articles (n = 73) (Table 4). The five most common methods used were reported as down-sampling (n = 16; 11%), Synthetic Majority Over Sampling (SMOTE) (n = 16; 11%), matched instances (n = 15; 10.3%), up-sampling (n = 9; 6.2%) and random sampling (n = 7; 4.8%).

#### Data preparation

Data preparation methods were not described in 79 articles (54.5%) (Table 4). Scaling/normalisation of the numerical variables was reported in 24 articles (16.6%), one-hot encoding of categorical variables was described in 19 articles (13.1%), and text-specific data preparation was reported in 17 articles (11.7%).

#### Missing data imputation

Methods used to impute missing data were not described in 61 articles (42.1%) (Table 4). In 24 articles (16.6%) missing data were reportedly removed from the dataset. In 16 articles (11.0%) mean imputation was reportedly performed for numerical variables. Median imputation was reported in eight articles (5.5%).

#### Choice of software

In most articles, ML models were derived using Python (n = 65, 44.8%) and R (n = 35; 24.1%) software. In 33 articles (22.8%), the software used was not reported (Table 4). Python software seems to be increasingly used followed by R (Supplemental Figures 1 and 2).

### Type of data and characteristics of the populations

Table 5 lists the main characteristics of the data sources used to derive EML models. Most data sources were composed of records from either secondary or tertiary healthcare facilities (n = 64, 44.1%), healthcare systems encompassing primary, secondary and tertiary care (n = 36, 24.8%), and primary care databases (n = 21; 14.5%). EHRs from tertiary facilities and registries were each reported in 10 articles (6.9%). Survey data were used in six (4.1%) of the articles. Seven articles (4.8%) did not clearly describe the level of care of their data source. 48.3% of articles used a data source made exclusively of adult patients (n = 70). 33.1% of articles did not clearly specify the age group (n = 48). The data source included both children and adults in 15 articles (10.3%). 12 articles (8.3%) reported EMLs derived from datasets composed solely of children patients.

**Table 5.** Characteristics of the data sources for ensemble models.

Property	Property value (top 10 values only)	*N* articles (%) or median (IQR)
Healthcare Settings	Described	138 (95.2)
	➔Secondary and/or tertiary care	64 (44.1)
	➔Primary, secondary and/or tertiary care	36 (24.8)
	➔Primary care	21 (14.5)
	➔Registry	10 (6.9)
	➔Tertiary care	10 (6.9)
	➔Survey	6 (4.1)
	➔Trial	1 (0.7)
	➔Biobank	1 (0.7)
	Not described	7 (4.8)
Age Group	Adults	70 (48.3)
	➔Any adults (> 18 years)	47 (32.4)
	➔Elderly (> 65 years)	19 (13.1)
	➔Middle-aged adults (35 to < 65 years)	16 (11.0)
	➔Young adults (18 to < 35 years)	4 (2.8)
	Not specified	48 (33.1)
	Both adults and children together	15 (10.3)
	Children (< 18 years)	12 (8.3)
	➔Any children	7 (4.8)
	➔Newborn	4 (2.8)
	➔Teenagers	1 (0.7)
Modality	Structured EHR only	121 (83.4)
	Structured and unstructured EHR	15 (10.3)
	Unstructured EHR only	9 (6.2)
Sample sizes	Training dataset	10,208 (1685–90,324); 68.13%
	Validation dataset	3838 (532–29,004); 22.62%
	Test dataset	5365 (956–43,038); 26.85%
Case imbalance	Modest imbalance [1%–20% and 80%–99%]	76 (52.4)
	Marginal imbalance [20%–40% and 60%–80%]	34 (23.4)
	Extreme imbalance [0%–1% and 99%–100%]	13 (9.0)
	No imbalance [40%–60%]	11 (7.6)
	Prevalence is missing	11 (7.6)
Number of predictors	Described	116 (80.0)
	➔0–50	51 (35.2)
	➔51–100	27 (18.6)
	➔101–1000	20 (13.8)
	➔>1000	18 (12.4)
	Not Described	17 (11.7)
	Text Format	12 (8.3)
Number of observations/patients per predictor	Training Set	106 [19–1036]
	Validation Set	28 [6–433]
	Test Set	98 [9–643]
Outcome definition	Described	122 (84.1)
	➔ICD code	66 (45.5)
	➔Chart review/in-person (i.e. clinician input)	34 (23.4)
	➔Clinical definition (criteria/guideline)	33 (22.8)
	➔Self-reported	5 (3.4)
	➔Read codes	4 (2.8)
	➔Medications	3 (2.1)
	➔SNOMED	2 (1.4)
	➔Procedure code	1 (0.7)
	Not described	23 (15.9)

Articles reported the derivation of EMLs from structured records (n = 121, 83.4), semi-structured records (n = 15, 10.3%), and unstructured records (n = 9; 6.2%). The sample size considerably varied between training (median: 10,208), validation (median: 3858) and test datasets (median: 5365).

Most training datasets reported were modestly imbalanced or marginally imbalanced (n = 34, 23.4% and n = 76, 52.4%, respectively). 13 articles (9%) had an extremely imbalanced dataset, whereas 11 articles did not report on class-imbalance (7.6%) or reported a balanced dataset (n = 11; 7.6%).

Authors included a varying number of predictors in their analyses or deriving models, ranging from less than 50 input variables (35.2% of articles, n = 51), to over 1000 predictors (12.4% of articles, n = 18) (Table 5). 11.7% of articles (n = 17) did not report the number of predictors.

The outcomes were mostly defined based on the ICD coding system (version 9 or 10) (n = 66, 45.5%) but also based on the expert review (either chart review or in-person tests) (n = 34, 23.4%). In 15.9% of articles (n = 23), the outcome was not adequately described. In 22.8% of articles (n = 33), the outcomes were derived by applying clinical definitions automatically such as clinical criteria, risk scores, or clinical guidelines.

### Adherence to TRIPOD guideline

Only 9% (n = 13) of articles report using the TRIPOD checklist. Reporting in accordance with the TRIPOD statement was more common in articles where the R software was used to derive EML (n = 7/35, 20%) compared to Python (n = 4/65, 6.2%) (two sample proportion Z-test *p*-value: 0.038).

## Discussion

This systematic scoping review summarises the field of EMLs applied to pre-screening EHRs to identify undiagnosed individuals.

### Application of EMLs

EML methods are increasingly being used in EHRs for pre-screening across all major medical specialties which is consistent with the findings of Yang et al.^22^ EMLs are likely to be more robust performing models, which is consistent with general beliefs about the superiority of EML over traditional ML methods.^23,24^ Parallel EML using a majority vote fusion strategy, such as Random Forest models had the lowest chances of being selected as the best models but were one of the most used EMLs. The statement of Hossain et al. and the finding of Nwanosike et al. that the Random Forest are one of the most accurate ML-based algorithms should thus, therefore, be considered with caution as we found that Random Forest models had less chances to be selected as the best models by studies’ authors.^23,24^ Conversely, we found that parallel EMLs using either average probabilities fusion, weighted vote, or stacking, or with heterogeneous base learners had the highest chances of being selected as best models but were the least commonly used EMLs. This observation highlights the importance of combining the predictions of heterogeneous bases learner, which are less likely to be correlated, than predictions originating from homogeneous base learners. Furthermore, it shows that more complex fusion strategies such as weighted average fusion, average fusion and stacking yield better predictive performances. In papers reporting the use of stacking ensembles, the meta-learners and data partitioning strategies were not reported clearly as reported.

### Pre-processing steps

Hyperparameter tuning is an important step to ensure that ML algorithms capture all the relationships present in the data when deriving a model without overfitting; yet it was often not described and/or underutilized, sometimes even used partly (which could hinder fair comparison between ML models derived from different algorithms).

A Plethora of feature selection methods have been used, including ensemble-based algorithms (e.g. Random Forest); however, most articles did not describe feature selection altogether.

The metrics used for the evaluation and comparison of models’ performances were mostly reported appropriately. All the studies included classification metrics as screening is inherently a classification task.

Most articles used a combination of cross-validation and held-out samples which is recommended to provide an unbiased estimation of the model performances. The overall data partitioning strategies were documented in most articles although not always very clearly. For example, most articles do not clarify whether cross-validation was used for hyperparameter tuning or reporting the final models’ performances.

Around 60% of articles had a moderate to extreme class imbalance but in a large proportion of them (43%), this issue was not acknowledged or appropriately addressed, which can severely impact the performances of ML models.

Approximately 50% of articles did not adequately describe data preparation or handling of missing data. This can hinder reproducibility but also lead to a significant loss of observations wherever missing values are prevalent such as in EHRs and thus prevent case finding. Some authors report that EML made of decision trees are particularly well suited to manage missing data, which is one of the advantages of EMLs.

Most articles appropriately reported the software environment used to derive their models.

### Data characteristics

Like in any other analysis, EMLs should be derived or at least tested on datasets representative of the target populations. EMLs are expected to retain good predictive performances when applied to samples drawn from similar populations. However, in most articles, the target populations were not clearly defined, and therefore it is difficult to assess whether models have been derived and tested on an appropriate dataset. While the type of data source used to derive models (i.e. the training population) was described nearly in all articles, it is not necessarily possible to assume that training and target populations are similar given the lack of transparency in the studies.

The authors of the reviewed articles ambitiously inferred that their ML models derived within a specific population could be applied to other populations; however, the limitation of their models, including descriptions of the populations used to derive the model, or where the model is intended to be used, should be well described in their articles. Secondary care datasets (i.e. specialised/emergency hospitals) were often employed to derive models intended to screen for common diseases in primary care EHRs, which is not uncommon due to the low prevalence of certain diseases in the general population. Similarly, the likelihood of having a disease may increase with age, therefore most reviewed articles reported the derivations of EMLs in older individuals, but the age of the population where such models would be used were not consistently given.

Most reviewed articles provide the number of predictors considered for inclusion in their models and their authors took advantage of the plethora of data available within EHRs by using more than 50 variables, sometimes even more than 100 or 1000 variables. Cases were more often defined using the ICD coding systems, chart review and in-person clinical assessments.

### Adherence to the tripod statement

In most articles reviewed in our study, authors did not follow the reporting guideline to ensure that their studies are at the required standards and quality. Guidelines such as the TRIPOD statement may considerably improve the transparency and reproducibility of a published model, leading to a higher chance of implementation in daily clinical practice. Although, as highlighted by Yang et al., the TRIPOD statement provides limited guidance on how to present the final model for non-regression models such as EMLs.^22,27^ The users of R statistical software/environment were more likely to refer to the TRIPOD statement than Python users, which may indicate a difference in education, training or reporting standards between Python and R users.

### Limitation

Some limitations of the present scoping review must be acknowledged. Firstly, the article selection and extraction have been performed by a single reviewer; however, we consider this case to be acceptable based on the nature and intentions of a scoping review study. Secondly, the important concepts of explainability and fairness of EMLs were out of the remit of this review due to the lack of information available on explainability in the literature and the complexity of assessing fairness. Future research is needed to assess the explainability and fairness of EML and how these concepts impact clinical practice. Thirdly, our conclusions based on the retrieved published articles do not necessarily represent a formal benchmarking of EMLs algorithms, which should be considered during the interpretation of the results.

### Conclusion

EML methods are increasingly being adopted in medical pre-screening of EHRs, which can have a significant impact on public health due to their ability to identify undiagnosed individuals with a potential disease with more sensitivity and specificity than non-ensemble models. EMLs with the highest performances, such as heterogeneous EMLs or stacking/weighted average fusion EMLs, are used to a lesser extent than EMLs with more modest performances such as homogeneous and majority voting fusions EMLs.

Further adoption of EML in the pre-screening of EHRs could be improved by enhancing the transparency, quality and reproducibility of studies reporting EMLs; this aspect could be accomplished through the provision of detailed descriptions by studies’ authors of their employed ML methods, preprocessing steps, data sources and populations where the model is intended to be used.

## Supplemental Material

sj-docx-1-dhj-10.1177_20552076231173225 - Supplemental material for Ensemble machine learning methods in screening electronic health records: A scoping review Click here for additional data file. Supplemental material, sj-docx-1-dhj-10.1177_20552076231173225 for Ensemble machine learning methods in screening electronic health records: A scoping review by Christophe AT Stevens, Alexander RM Lyons, Kanika I Dharmayat, Alireza Mahani, Kausik K Ray, Antonio J Vallejo-Vaz and Mansour TA Sharabiani in DIGITAL HEALTH

sj-docx-2-dhj-10.1177_20552076231173225 - Supplemental material for Ensemble machine learning methods in screening electronic health records: A scoping review Click here for additional data file. Supplemental material, sj-docx-2-dhj-10.1177_20552076231173225 for Ensemble machine learning methods in screening electronic health records: A scoping review by Christophe AT Stevens, Alexander RM Lyons, Kanika I Dharmayat, Alireza Mahani, Kausik K Ray, Antonio J Vallejo-Vaz and Mansour TA Sharabiani in DIGITAL HEALTH

sj-docx-3-dhj-10.1177_20552076231173225 - Supplemental material for Ensemble machine learning methods in screening electronic health records: A scoping review Click here for additional data file. Supplemental material, sj-docx-3-dhj-10.1177_20552076231173225 for Ensemble machine learning methods in screening electronic health records: A scoping review by Christophe AT Stevens, Alexander RM Lyons, Kanika I Dharmayat, Alireza Mahani, Kausik K Ray, Antonio J Vallejo-Vaz and Mansour TA Sharabiani in DIGITAL HEALTH

sj-docx-4-dhj-10.1177_20552076231173225 - Supplemental material for Ensemble machine learning methods in screening electronic health records: A scoping review Click here for additional data file. Supplemental material, sj-docx-4-dhj-10.1177_20552076231173225 for Ensemble machine learning methods in screening electronic health records: A scoping review by Christophe AT Stevens, Alexander RM Lyons, Kanika I Dharmayat, Alireza Mahani, Kausik K Ray, Antonio J Vallejo-Vaz and Mansour TA Sharabiani in DIGITAL HEALTH

sj-docx-5-dhj-10.1177_20552076231173225 - Supplemental material for Ensemble machine learning methods in screening electronic health records: A scoping review Click here for additional data file. Supplemental material, sj-docx-5-dhj-10.1177_20552076231173225 for Ensemble machine learning methods in screening electronic health records: A scoping review by Christophe AT Stevens, Alexander RM Lyons, Kanika I Dharmayat, Alireza Mahani, Kausik K Ray, Antonio J Vallejo-Vaz and Mansour TA Sharabiani in DIGITAL HEALTH

sj-docx-6-dhj-10.1177_20552076231173225 - Supplemental material for Ensemble machine learning methods in screening electronic health records: A scoping review Click here for additional data file. Supplemental material, sj-docx-6-dhj-10.1177_20552076231173225 for Ensemble machine learning methods in screening electronic health records: A scoping review by Christophe AT Stevens, Alexander RM Lyons, Kanika I Dharmayat, Alireza Mahani, Kausik K Ray, Antonio J Vallejo-Vaz and Mansour TA Sharabiani in DIGITAL HEALTH

sj-docx-7-dhj-10.1177_20552076231173225 - Supplemental material for Ensemble machine learning methods in screening electronic health records: A scoping review Click here for additional data file. Supplemental material, sj-docx-7-dhj-10.1177_20552076231173225 for Ensemble machine learning methods in screening electronic health records: A scoping review by Christophe AT Stevens, Alexander RM Lyons, Kanika I Dharmayat, Alireza Mahani, Kausik K Ray, Antonio J Vallejo-Vaz and Mansour TA Sharabiani in DIGITAL HEALTH

sj-docx-8-dhj-10.1177_20552076231173225 - Supplemental material for Ensemble machine learning methods in screening electronic health records: A scoping review Click here for additional data file. Supplemental material, sj-docx-8-dhj-10.1177_20552076231173225 for Ensemble machine learning methods in screening electronic health records: A scoping review by Christophe AT Stevens, Alexander RM Lyons, Kanika I Dharmayat, Alireza Mahani, Kausik K Ray, Antonio J Vallejo-Vaz and Mansour TA Sharabiani in DIGITAL HEALTH
