# Supplementary material for: Ensemble machine learning methods in screening electronic health records: A scoping review
Source: Digit Health. 2023 May 9;9:20552076231173225. doi: 10.1177/20552076231173225 (PMC10176785; doi:10.1177/20552076231173225)
Supplement: sj-docx-2-dhj-10.1177_20552076231173225 - Supplemental material for Ensemble machine learning methods in screening electronic health records: A scoping review [file sj-docx-2-dhj-10.1177_20552076231173225.docx]

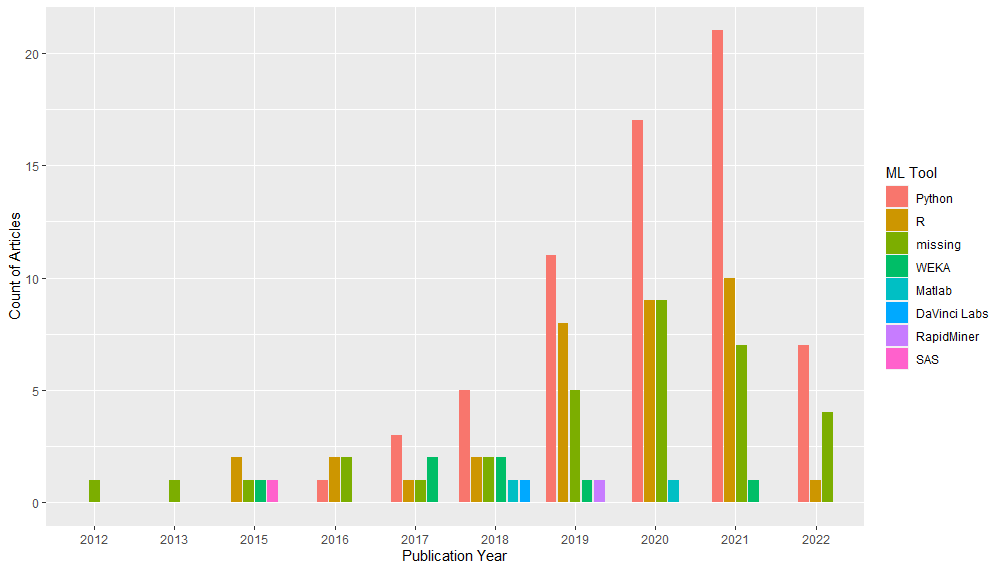


Supplemental Figure 1: Count of articles reporting the use of software for the derivation of EML by year*.

*The search strategy was until April 2022 and thus the graph does not show the full year.
