# Supplementary figures and images for: Ensemble machine learning methods in screening electronic health records: A scoping review
Source: Digit Health. 2023 May 9;9:20552076231173225. doi: 10.1177/20552076231173225 (PMC10176785; doi:10.1177/20552076231173225)

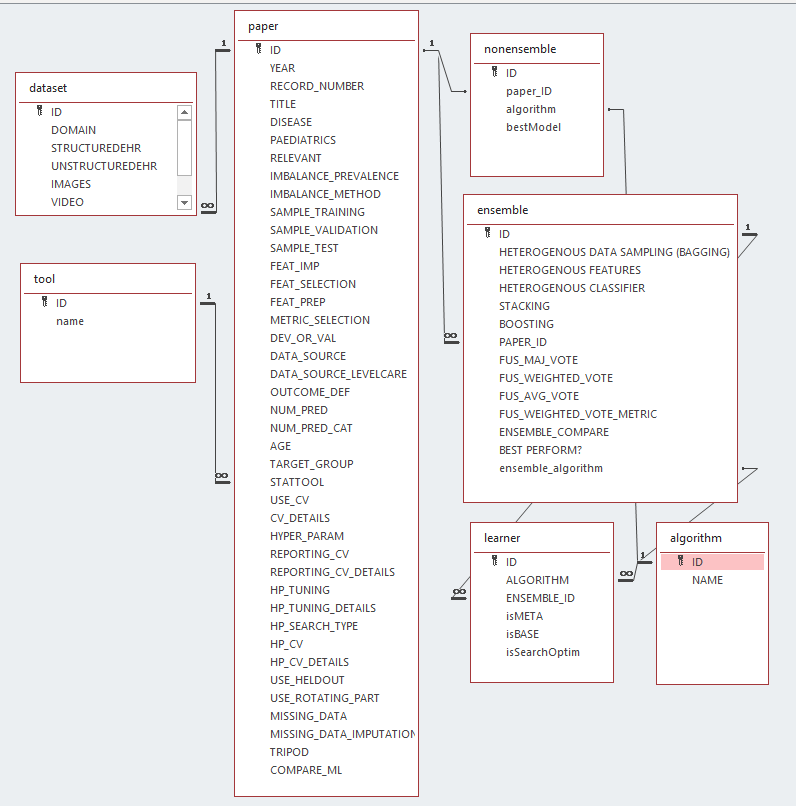


Supplemental Figure 3. Diagram of the Microsoft Access database and its collected fields.

Supplement: sj-docx-4-dhj-10.1177_20552076231173225 - Supplemental material for Ensemble machine learning methods in screening electronic health records: A scoping review [file sj-docx-4-dhj-10.1177_20552076231173225.docx]
