# Supplementary material for: Ensemble machine learning methods in screening electronic health records: A scoping review
Source: Digit Health. 2023 May 9;9:20552076231173225. doi: 10.1177/20552076231173225 (PMC10176785; doi:10.1177/20552076231173225)
Supplement: sj-docx-5-dhj-10.1177_20552076231173225 - Supplemental material for Ensemble machine learning methods in screening electronic health records: A scoping review [file sj-docx-5-dhj-10.1177_20552076231173225.docx]

**Supplemental Reference 1: Articles reporting EML for medical screening of EHRs, included in the analysis.**

[1-145]

[1] M. Abdalla *et al.*, "Accuracy of Algorithms to Identify People with Atopic Dermatitis in Ontario Routinely Collected Health Databases," (in English), *Journal of Investigative Dermatology,* vol. 141, no. 7, pp. 1840-1843, 2021, doi: <http://dx.doi.org/10.1016/j.jid.2021.01.009>.

[2] Y. Yang, J. Zheng, Z. Du, Y. Li, and Y. Cai, "Accurate Prediction of Stroke for Hypertensive Patients Based on Medical Big Data and Machine Learning Algorithms: Retrospective Study," *JMIR medical informatics,* vol. 9, no. 11, p. e30277, 2021, doi: <https://dx.doi.org/10.2196/30277>.

[3] J. S. Obeid, A. Khalifa, B. Xavier, H. Bou-Daher, and D. C. Rockey, "An AI Approach for Identifying Patients with Cirrhosis," (in English), *Journal of Clinical Gastroenterology,* 2021, doi: <http://dx.doi.org/10.1097/MCG.0000000000001586>.

[4] H. Zafari, S. Langlois, F. Zulkernine, L. Kosowan, and A. Singer, "AI in predicting COPD in the Canadian population," *Bio Systems,* vol. 211, p. 104585, 2022, doi: <https://dx.doi.org/10.1016/j.biosystems.2021.104585>.

[5] J.-G. Shim *et al.*, "Application of machine learning approaches for osteoporosis risk prediction in postmenopausal women," (in English), *Archives of Osteoporosis,* vol. 15, no. 1, p. 169, 2020, doi: <http://dx.doi.org/10.1007/s11657-020-00802-8>.

[6] S. Fialoke, A. Malarstig, M. R. Miller, and A. Dumitriu, "Application of Machine Learning Methods to Predict Non-Alcoholic Steatohepatitis (NASH) in Non-Alcoholic Fatty Liver (NAFL) Patients," (in English), *AMIA ... Annual Symposium proceedings. AMIA Symposium,* vol. 2018, pp. 430-439, 2018. [Online]. Available: <http://ovidsp.ovid.com/ovidweb.cgi?T=JS&PAGE=reference&D=emed19&NEWS=N&AN=626622046//https://www.ncbi.nlm.nih.gov/pmc/articles/PMC6371264/pdf/2974699.pdf>.

[7] P. Tiwari, K. L. Colborn, F. Xing, D. Ghosh, D. E. Smith, and M. A. Rosenberg, "Assessment of a Machine Learning Model Applied to Harmonized Electronic Health Record Data for the Prediction of Incident Atrial Fibrillation," (in English), *JAMA Network Open,* vol. 3, no. 1, p. 19396, 2020, doi: <http://dx.doi.org/10.1001/jamanetworkopen.2019.19396>.

[8] S. G.-. Murray, J.-. -Yazdany, A.-. -Avati, and G. -Schmajuk, "Automated and flexible identification of complex disease: Building a model for systemic lupus erythematosus using noisy labeling," (in English), *Journal of the American Medical Informatics Association,* vol. 26, no. 1, pp. 61-65, 2019, doi: <http://dx.doi.org/10.1093/jamia/ocy154>.

[9] E. Ford, J. A. Cassell, S. Oliver, P. Rooney, J. Sheppard, and S. Banerjee, "Automated detection of patients with dementia whose symptoms have been identified in primary care but have no formal diagnosis: A retrospective case-control study using electronic primary care records," (in English), *BMJ Open,* vol. 11, no. 1, p. e039248, 2021, doi: <http://dx.doi.org/10.1136/bmjopen-2020-039248>.

[10] L. N. Dalbonio de Carvalho *et al.*, "Can machine learning be useful as a screening tool for depression in primary care?," (in English), *Journal of Psychiatric Research,* vol. 132, pp. 1-6, 2021, doi: <http://dx.doi.org/10.1016/j.jpsychires.2020.09.025>.

[11] A. Malhotra, B. Rachet, L. M. Woods, A. Bonaventure, and S. P. Pereira, "Can we screen for pancreatic cancer? Identifying a sub-population of patients at high risk of subsequent diagnosis using machine learning techniques applied to primary care data," (in English), *PLoS ONE,* vol. 16, no. 6 June, p. e0251876, 2021, doi: <http://dx.doi.org/10.1371/journal.pone.0251876>.

[12] L. C.-. Costa *et al.*, "Classification algorithm for congenital Zika Syndrome: characterizations, diagnosis and validation," (in English), *Scientific reports,* vol. 11, no. 1, p. 6770, 2021, doi: <http://dx.doi.org/10.1038/s41598-021-86361-5>.

[13] T. B.-. Nguyen *et al.*, "Classification of suicide attempters in schizophrenia using sociocultural and clinical features: A machine learning approach," (in English), *General Hospital Psychiatry,* vol. 47, pp. 20-28, 2017, doi: <http://dx.doi.org/10.1016/j.genhosppsych.2017.03.001>.

[14] Q. Chen *et al.*, "Clinical Data Prediction Model to Identify Patients With Early-Stage Pancreatic Cancer," (in English), *JCO clinical cancer informatics,* vol. 5, pp. 279-287, 2021, doi: <http://dx.doi.org/10.1200/CCI.20.00137>.

[15] H. Li *et al.*, "Colorectal Cancer Detected by Machine Learning Models Using Conventional Laboratory Test Data," (in English), *Technology in cancer research & treatment,* vol. 20, p. 15330338211058352, 2021, doi: <https://dx.doi.org/10.1177/15330338211058352>.

[16] P. Fraccaro *et al.*, "Combining macula clinical signs and patient characteristics for age-related macular degeneration diagnosis: a machine learning approach," (in English), *BMC ophthalmology,* vol. 15, p. 10, 2015, doi: <http://dx.doi.org/10.1186/1471-2415-15-10>.

[17] P. M. Thangaraj, T. Lorberbaum, N. P. Tatonetti, B. R. Kummer, and M. S. V. Elkind, "Comparative analysis, applications, and interpretation of electronic health record-based stroke phenotyping methods," (in English), *BioData Mining,* vol. 13, no. 1, p. 21, 2020, doi: <http://dx.doi.org/10.1186/s13040-020-00230-x>.

[18] J.-. Parreco *et al.*, "Comparing machine learning algorithms for predicting acute kidney injury," (in English), *American Surgeon,* vol. 85, no. 7, pp. 725-729, 2019, doi: <http://dx.doi.org/10.1177/000313481908500731>.

[19] A.-. Lopez Pineda, Y.-. -Ye, S.-. -Visweswaran, G. F.-. -Cooper, M. M.-. -Wagner, and F. -Rich Tsui, "Comparison of machine learning classifiers for influenza detection from emergency department free-text reports," (in English), *Journal of Biomedical Informatics,* vol. 58, pp. 60-69, 2015, doi: <http://dx.doi.org/10.1016/j.jbi.2015.08.019>.

[20] H.-. Zhu, J.-. -Yao, Q.-. -Gao, X.-. -Xia, H.-. -Fan, and Q. -Wang, "Comparisons of different classification algorithms while using text mining to screen psychiatric inpatients with suicidal behaviors," (in English), *Journal of Psychiatric Research,* vol. 124, pp. 123-130, 2020, doi: <http://dx.doi.org/10.1016/j.jpsychires.2020.02.019>.

[21] D. Chicco and C. Rovelli, "Computational prediction of diagnosis and feature selection on mesothelioma patient health records," (in English), *PLoS ONE,* vol. 14, no. 1, p. e0208737, 2019, doi: <http://dx.doi.org/10.1371/journal.pone.0208737>.

[22] E. Ford *et al.*, "Could dementia be detected from UK primary care patients' records by simple automated methods earlier than by the treating physician? A retrospective case-control study," *Wellcome open research,* vol. 5, p. 120, 2020, doi: <https://dx.doi.org/10.12688/wellcomeopenres.15903.1>.

[23] A. Dinh, S. Miertschin, A. Young, and S. D. Mohanty, "A data-driven approach to predicting diabetes and cardiovascular disease with machine learning," (in English), *BMC medical informatics and decision making,* vol. 19, no. 1, p. 211, 2019, doi: <http://dx.doi.org/10.1186/s12911-019-0918-5>.

[24] V. S.-. Nori, C. A.-. -Hane, Y.-. -Sun, W. H.-. -Crown, and P. A. -Bleicher, "Deep neural network models for identifying incident dementia using claims and EHR datasets," (in English), *PLoS ONE,* vol. 15, no. 9 September, p. e0236400, 2020, doi: <http://dx.doi.org/10.1371/journal.pone.0236400>.

[25] O. I. Ogunyemi *et al.*, "Detecting diabetic retinopathy through machine learning on electronic health record data from an urban, safety net healthcare system," *JAMIA open,* vol. 4, no. 3, p. ooab066, 2021, doi: <https://dx.doi.org/10.1093/jamiaopen/ooab066>.

[26] G. Liu *et al.*, "Developing a Machine Learning System for Identification of Severe Hand, Foot, and Mouth Disease from Electronic Medical Record Data," (in English), *Scientific reports,* vol. 7, no. 1, p. 16341, 2017, doi: <http://dx.doi.org/10.1038/s41598-017-16521-z>.

[27] A. Barnado *et al.*, "Developing and Validating Methods to Assemble Systemic Lupus Erythematosus Births in the Electronic Health Record," (in English), *Arthritis care & research,* 2020, doi: <http://dx.doi.org/10.1002/acr.24522>.

[28] K.-C.-. Yuan *et al.*, "The development an artificial intelligence algorithm for early sepsis diagnosis in the intensive care unit," (in English), *International Journal of Medical Informatics,* vol. 141, p. 104176, 2020, doi: <http://dx.doi.org/10.1016/j.ijmedinf.2020.104176>.

[29] R. J.-. Delahanty, J.-. -Alvarez, L. M.-. -Flynn, S. S.-. -Jones, and R. L. -Sherwin, "Development and Evaluation of a Machine Learning Model for the Early Identification of Patients at Risk for Sepsis," (in English), *Annals of Emergency Medicine,* vol. 73, no. 4, pp. 334-344, 2019, doi: <http://dx.doi.org/10.1016/j.annemergmed.2018.11.036>.

[30] S. Wang, Y. Zhang, A. Hermann, J. Pathak, and R. Joly, "Development and validation of a machine learning algorithm for predicting the risk of postpartum depression among pregnant women," (in English), *Journal of Affective Disorders,* vol. 279, pp. 1-8, 2021, doi: <http://dx.doi.org/10.1016/j.jad.2020.09.113>.

[31] S. V.-. Bhavani *et al.*, "The development and validation of a machine learning model to predict bacteremia and fungemia in hospitalized patients using electronic health record data," (in English), *Critical Care Medicine,* pp. E1020-E1028, 2020, doi: <http://dx.doi.org/10.1097/CCM.0000000000004556>.

[32] S. Gur *et al.*, "Development and validation of a machine learning-based postpartum depression prediction model: A nationwide cohort study," (in English), *Depression and Anxiety,* vol. 38, no. 4, pp. 400-411, 2021, doi: <http://dx.doi.org/10.1002/da.23123>.

[33] Y. Kinar *et al.*, "Development and validation of a predictive model for detection of colorectal cancer in primary care by analysis of complete blood counts: A binational retrospective study," (in English), *Journal of the American Medical Informatics Association,* vol. 23, no. 5, pp. 879-890, 2016, doi: <http://dx.doi.org/10.1093/jamia/ocv195>.

[34] E. M. Heifetz *et al.*, "Development and validation of a risk prediction model to diagnose Barrett's oesophagus (MARK-BE): a case-control machine learning approach," (in English), *The Lancet Digital Health,* vol. 2, no. 1, pp. e37-e48, 2020, doi: <http://dx.doi.org/10.1016/S2589-7500%2819%2930216-X>.

[35] L.-. Ehwerhemuepha *et al.*, "Development and validation of an early warning tool for sepsis and decompensation in children during emergency department triage," (in English), *Scientific reports,* vol. 11, no. 1, p. 8578, 2021, doi: <http://dx.doi.org/10.1038/s41598-021-87595-z>.

[36] M. Docherty *et al.*, "Development of a novel machine learning model to predict presence of nonalcoholic steatohepatitis," (in English), *Journal of the American Medical Informatics Association,* vol. 28, no. 6, pp. 1235-1241, 2021, doi: <http://dx.doi.org/10.1093/jamia/ocab003>.

[37] J. D. Koola *et al.*, "Development of an automated phenotyping algorithm for hepatorenal syndrome," (in English), *Journal of Biomedical Informatics,* vol. 80, pp. 87-95, 2018, doi: <http://dx.doi.org/10.1016/j.jbi.2018.03.001>.

[38] L.-. Zheng *et al.*, "Development of an early-warning system for high-risk patients for suicide attempt using deep learning and electronic health records," (in English), *Translational Psychiatry,* vol. 10, no. 1, p. 72, 2020, doi: <http://dx.doi.org/10.1038/s41398-020-0684-2>.

[39] H.-. Hegde, N.-. -Shimpi, A.-. -Panny, I.-. -Glurich, P.-. -Christie, and A. -Acharya, "Development of non-invasive diabetes risk prediction models as decision support tools designed for application in the dental clinical environment," (in English), *Informatics in Medicine Unlocked,* vol. 17, p. 100254, 2019, doi: <http://dx.doi.org/10.1016/j.imu.2019.100254>.

[40] J. Shin *et al.*, "Development of Various Diabetes Prediction Models Using Machine Learning Techniques," (in English), *Diabetes & metabolism journal,* 2022, doi: <https://dx.doi.org/10.4093/dmj.2021.0115>.

[41] E. Oh, T. K. Yoo, and E.-C. Park, "Diabetic retinopathy risk prediction for fundus examination using sparse learning: a cross-sectional study," (in English), *BMC medical informatics and decision making,* vol. 13, p. 106, 2013. [Online]. Available: <http://ovidsp.ovid.com/ovidweb.cgi?T=JS&PAGE=reference&D=emexb&NEWS=N&AN=563075765//https://www.ncbi.nlm.nih.gov/pmc/articles/PMC3847617/pdf/1472-6947-13-106.pdf>.

[42] H. Zafari, L. Kosowan, F. Zulkernine, and A. Signer, "Diagnosing post-traumatic stress disorder using electronic medical record data," (in English), *Health informatics journal,* vol. 27, no. 4, p. 14604582211053259, 2021, doi: <https://dx.doi.org/10.1177/14604582211053259>.

[43] Y. Li, S. Tian, W. Dong, and Y. Huang, "Driverless artificial intelligence framework for the identification of malignant pleural effusion," (in English), *Translational Oncology,* vol. 14, no. 1, p. 100896, 2021, doi: <http://dx.doi.org/10.1016/j.tranon.2020.100896>.

[44] K. Ng, S. R. Steinhubl, C. Defilippi, S. Dey, and W. F. Stewart, "Early Detection of Heart Failure Using Electronic Health Records: Practical Implications for Time before Diagnosis, Data Diversity, Data Quantity, and Data Density," (in English), *Circulation: Cardiovascular Quality and Outcomes,* vol. 9, no. 6, pp. 649-658, 2016, doi: <http://dx.doi.org/10.1161/CIRCOUTCOMES.116.002797>.

[45] M. E.-. Kalor *et al.*, "Early detection of sepsis utilizing deep learning on electronic health record event sequences," (in English), *Artificial Intelligence in Medicine,* vol. 104, p. 101820, 2020, doi: <http://dx.doi.org/10.1016/j.artmed.2020.101820>.

[46] J. S.-. Hinson *et al.*, "Early Prediction of Acute Kidney Injury in the Emergency Department With Machine-Learning Methods Applied to Electronic Health Record Data," (in English), *Annals of Emergency Medicine,* vol. 76, no. 4, pp. 501-514, 2020, doi: <http://dx.doi.org/10.1016/j.annemergmed.2020.05.026>.

[47] A. Abdullah Alfayez, A. Grace Lai, and H. Kunz, "Early Prediction of Neoplasms Using Machine Learning: A Study of Electronic Health Records from the Ministry of National Guard Health Affairs in Saudi Arabia," (in English), *Studies in health technology and informatics,* vol. 289, pp. 37-40, 2022, doi: <https://dx.doi.org/10.3233/SHTI210853>.

[48] I. Maric *et al.*, "Early prediction of preeclampsia via machine learning," (in English), *American Journal of Obstetrics and Gynecology MFM,* vol. 2, no. 2, p. 100100, 2020, doi: <http://dx.doi.org/10.1016/j.ajogmf.2020.100100>.

[49] M.-. Saqib, Y.-. -Sha, and M. D. -Wang, "Early Prediction of Sepsis in EMR Records Using Traditional ML Techniques and Deep Learning LSTM Networks," (in English), *Conference proceedings : ... Annual International Conference of the IEEE Engineering in Medicine and Biology Society. IEEE Engineering in Medicine and Biology Society. Annual Conference,* vol. 2018, pp. 4038-4041, 2018, doi: <http://dx.doi.org/10.1109/EMBC.2018.8513254>.

[50] R. Thapa *et al.*, "Early prediction of severe acute pancreatitis using machine learning," (in English), *Pancreatology,* vol. 22, no. 1, pp. 43-50, 2022, doi: <https://dx.doi.org/10.1016/j.pan.2021.10.003>.

[51] M. Bernardini, M. Morettini, E. Frontoni, L. Burattini, and L. Romeo, "Early temporal prediction of Type 2 Diabetes Risk Condition from a General Practitioner Electronic Health Record: A Multiple Instance Boosting Approach," (in English), *Artificial Intelligence in Medicine,* vol. 105, p. 101847, 2020, doi: <http://dx.doi.org/10.1016/j.artmed.2020.101847>.

[52] A. N. Richter and T. M. Khoshgoftaar, "Efficient learning from big data for cancer risk modeling: A case study with melanoma," (in English), *Computers in Biology and Medicine,* vol. 110, pp. 29-39, 2019, doi: <http://dx.doi.org/10.1016/j.compbiomed.2019.04.039>.

[53] A. E. Anderson, A. Thames, T. Li, J. Xiao, W. T. Kerr, and M. S. Cohen, "Electronic health record phenotyping improves detection and screening of type 2 diabetes in the general United States population: A cross-sectional, unselected, retrospective study," (in English), *Journal of Biomedical Informatics,* vol. 60, pp. 162-168, 2016, doi: <http://dx.doi.org/10.1016/j.jbi.2015.12.006>.

[54] Y. Zhang *et al.*, "Electronic Health Record-Based Prediction of 1-Year Risk of Incident Cardiac Dysrhythmia: Prospective Case-Finding Algorithm Development and Validation Study," *JMIR medical informatics,* vol. 9, no. 2, p. e23606, 2021, doi: <https://dx.doi.org/10.2196/23606>.

[55] M. Nakayama and R. Inoue, "Electronic Phenotyping to Identify Patients with Heart Failure Using a National Clinical Information Database in Japan," (in English), *Studies in health technology and informatics,* vol. 281, pp. 243-247, 2021, doi: <http://dx.doi.org/10.3233/SHTI210157>.

[56] J. Maharjan *et al.*, "Enriching the Study Population for Ischemic Stroke Therapeutic Trials Using a Machine Learning Algorithm," (in English), *Frontiers in Neurology,* vol. 12, p. 784250, 2022, doi: <https://dx.doi.org/10.3389/fneur.2021.784250>.

[57] G. Amit *et al.*, "Estimation of postpartum depression risk from electronic health records using machine learning," (in English), *BMC Pregnancy and Childbirth,* vol. 21, no. 1, p. 630, 2021, doi: <http://dx.doi.org/10.1186/s12884-021-04087-8>.

[58] P. L. Teixeira *et al.*, "Evaluating electronic health record data sources and algorithmic approaches to identify hypertensive individuals," (in English), *Journal of the American Medical Informatics Association,* vol. 24, no. 1, pp. 162-171, 2017, doi: <http://dx.doi.org/10.1093/jamia/ocw071>.

[59] N. Z. Farahani, S. P. Arunachalam, D. S. B. Sundaram, K. Pasupathy, M. Enayati, and A. M. Arruda-Olson, "Explanatory Analysis of a Machine Learning Model to Identify Hypertrophic Cardiomyopathy Patients from EHR Using Diagnostic Codes," *Proceedings. IEEE International Conference on Bioinformatics and Biomedicine,* vol. 2020, pp. 1932-1937, 2020, doi: <https://dx.doi.org/10.1109/bibm49941.2020.9313231>.

[60] M. Zhao, T. Luo, C. Song, T. Huang, and S. Lin, "Fatty Liver Disease Prediction Model Based on Big Data of Electronic Physical Examination Records," (in English), *Frontiers in public health,* vol. 9, p. 668351, 2021, doi: <http://dx.doi.org/10.3389/fpubh.2021.668351>.

[61] S. Dubois *et al.*, "Finding missed cases of familial hypercholesterolemia in health systems using machine learning," (in English), *npj Digital Medicine,* vol. 2, no. 1, p. 23, 2019, doi: <http://dx.doi.org/10.1038/s41746-019-0101-5>.

[62] Y. Meng, W. Speier, M. Ong, and C. W. Arnold, "HCET: Hierarchical clinical embedding with topic modeling on electronic health records for predicting future depression," (in English), *IEEE Journal of Biomedical and Health Informatics,* vol. 25, no. 4, pp. 1265-1272, 2021, doi: <http://dx.doi.org/10.1109/JBHI.2020.3004072>.

[63] B.-. Emir, E. T.-. -Masters, J.-. -Mardekian, A.-. -Clair, M.-. -Kuhn, and S. L. -Silverman, "Identification of a potential fibromyalgia diagnosis using random forest modeling applied to electronic medical records," (in English), *Journal of Pain Research,* vol. 8, pp. 277-288, 2015, doi: <http://dx.doi.org/10.2147/JPR.S82566>.

[64] R. Rahman, A. Schlessinger, A. Kodesh, S. Z. Levine, S. Sandin, and A. Reichenberg, "Identification of newborns at risk for autism using electronic medical records and machine learning," (in English), *European psychiatry : the journal of the Association of European Psychiatrists,* vol. 63, no. 1, p. e22, 2020, doi: <http://dx.doi.org/10.1192/j.eurpsy.2020.17>.

[65] O. M. Doyle *et al.*, "Identification of potentially undiagnosed patients with nontuberculous mycobacterial lung disease using machine learning applied to primary care data in the UK," (in English), *European Respiratory Journal,* vol. 56, no. 4, p. 2000045, 2020, doi: <http://dx.doi.org/10.1183/13993003.00045-2020>.

[66] B.-. Mullin *et al.*, "Identification of suicidal behavior among psychiatrically hospitalized adolescents using natural language processing and machine learning of electronic health records," (in English), *PLoS ONE,* vol. 14, no. 2, p. e0211116, 2019, doi: <http://dx.doi.org/10.1371/journal.pone.0211116>.

[67] X. Yang *et al.*, "Identifying Cancer Patients at Risk for Heart Failure Using Machine Learning Methods," (in English), *AMIA ... Annual Symposium proceedings. AMIA Symposium,* vol. 2019, pp. 933-941, 2019. [Online]. Available: <http://ovidsp.ovid.com/ovidweb.cgi?T=JS&PAGE=reference&D=emed20&NEWS=N&AN=631583710//https://www.ncbi.nlm.nih.gov/pmc/articles/PMC7153053/pdf/3201850.pdf>.

[68] M. G. Seneviratne, J. M. Banda, N. H. Shah, T. M. Hernandez-Boussard, and J. D. Brooks, "Identifying Cases of Metastatic Prostate Cancer Using Machine Learning on Electronic Health Records," (in English), *AMIA ... Annual Symposium proceedings. AMIA Symposium,* vol. 2018, pp. 1498-1504, 2018. [Online]. Available: <http://ovidsp.ovid.com/ovidweb.cgi?T=JS&PAGE=reference&D=emed19&NEWS=N&AN=626622010//https://www.ncbi.nlm.nih.gov/pmc/articles/PMC6371284/pdf/2975482.pdf>.

[69] E. Ford *et al.*, "Identifying undetected dementia in UK primary care patients: a retrospective case-control study comparing machine-learning and standard epidemiological approaches," (in English), *BMC medical informatics and decision making,* vol. 19, no. 1, p. 248, 2019, doi: <http://dx.doi.org/10.1186/s12911-019-0991-9>.

[70] M.-. Bhattacharya *et al.*, "Identifying Ventricular Arrhythmias and Their Predictors by Applying Machine Learning Methods to Electronic Health Records in Patients With Hypertrophic Cardiomyopathy (HCM-VAr-Risk Model)," (in English), *American Journal of Cardiology,* vol. 123, no. 10, pp. 1681-1689, 2019, doi: <http://dx.doi.org/10.1016/j.amjcard.2019.02.022>.

[71] E.-. Molina, R.-. -Salazar-Cabrera, D. M.-. -Lopez, C. E. S.-. -Torres, and R. -Vargas-Canas, "Intelligent telehealth system to support epilepsy diagnosis," (in English), *Journal of Multidisciplinary Healthcare,* vol. 13, pp. 433-445, 2020, doi: <http://dx.doi.org/10.2147/JMDH.S247878>.

[72] O. Uspenskaya-Cadoz *et al.*, "Machine Learning Algorithm Helps Identify Non-Diagnosed Prodromal Alzheimer's Disease Patients in the General Population," (in English), *Journal of Prevention of Alzheimer's Disease,* vol. 6, no. 3, pp. 185-191, 2019, doi: <http://dx.doi.org/10.14283/jpad.2019.10>.

[73] H. M.-. Giannini *et al.*, "A Machine Learning Algorithm to Predict Severe Sepsis and Septic Shock: Development, Implementation, and Impact on Clinical Practice," (in English), *Critical care medicine,* vol. 47, no. 11, pp. 1485-1492, 2019, doi: <http://dx.doi.org/10.1097/CCM.0000000000003891>.

[74] N. Kijpaisalratana, D. Sanglertsinlapachai, S. Techaratsami, K. Musikatavorn, and J. Saoraya, "Machine learning algorithms for early sepsis detection in the emergency department: A retrospective study," (in English), *International Journal of Medical Informatics,* vol. 160, p. 104689, 2022, doi: <https://dx.doi.org/10.1016/j.ijmedinf.2022.104689>.

[75] L. N. Bonnell, B. Littenberg, G. L. Rose, and S. R. Wshah, "A machine learning approach to identification of unhealthy drinking," (in English), *Journal of the American Board of Family Medicine,* vol. 33, no. 3, pp. 397-406, 2020, doi: <http://dx.doi.org/10.3122/jabfm.2020.03.190421>.

[76] A. Sarraju, A. Ward, S. Chung, J. Li, D. Scheinker, and F. Rodriguez, "Machine learning approaches improve risk stratification for secondary cardiovascular disease prevention in multiethnic patients," (in English), *Open Heart,* vol. 8, no. 2, p. e001802, 2021, doi: <http://dx.doi.org/10.1136/openhrt-2021-001802>.

[77] T. D. Maarseveen *et al.*, "Machine Learning Electronic Health Record Identification of Patients with Rheumatoid Arthritis: Algorithm Pipeline Development and Validation Study," *JMIR medical informatics,* vol. 8, no. 11, p. e23930, 2020, doi: <https://dx.doi.org/10.2196/23930>.

[78] L.-. Xiang *et al.*, "Machine Learning for Early Warning of Septic Shock in Children With Hematological Malignancies Accompanied by Fever or Neutropenia: A Single Center Retrospective Study," (in English), *Frontiers in Oncology,* vol. 11, p. 678743, 2021, doi: <http://dx.doi.org/10.3389/fonc.2021.678743>.

[79] M.-. Bhattacharya *et al.*, "Machine Learning Methods for Identifying Atrial Fibrillation Cases and Their Predictors in Patients With Hypertrophic Cardiomyopathy: The HCM-AF-Risk Model," (in English), *CJC Open,* vol. 3, no. 6, pp. 801-813, 2021, doi: <http://dx.doi.org/10.1016/j.cjco.2021.01.016>.

[80] D. Wang *et al.*, "A Machine Learning Model for Accurate Prediction of Sepsis in ICU Patients," (in English), *Frontiers in public health,* vol. 9, p. 754348, 2021, doi: <https://dx.doi.org/10.3389/fpubh.2021.754348>.

[81] A. Huda *et al.*, "A machine learning model for identifying patients at risk for wild-type transthyretin amyloid cardiomyopathy," (in English), *Nature Communications,* vol. 12, no. 1, p. 2725, 2021, doi: <http://dx.doi.org/10.1038/s41467-021-22876-9>.

[82] R. G. Hauser *et al.*, "A Machine Learning Model to Successfully Predict Future Diagnosis of Chronic Myelogenous Leukemia With Retrospective Electronic Health Records Data," (in English), *American journal of clinical pathology,* 2021, doi: <http://dx.doi.org/10.1093/ajcp/aqab086>.

[83] A. J.-. Masino *et al.*, "Machine learning models for early sepsis recognition in the neonatal intensive care unit using readily available electronic health record data," (in English), *PLoS ONE,* vol. 14, no. 2, p. e0212665, 2019, doi: <http://dx.doi.org/10.1371/journal.pone.0212665>.

[84] J. Yu *et al.*, "Machine learning models for screening carotid atherosclerosis in asymptomatic adults," (in English), *Scientific reports,* vol. 11, no. 1, p. 22236, 2021, doi: <https://dx.doi.org/10.1038/s41598-021-01456-3>.

[85] J. H. Park *et al.*, "Machine learning prediction of incidence of Alzheimer's disease using large-scale administrative health data," (in English), *npj Digital Medicine,* vol. 3, no. 1, p. 46, 2020, doi: <http://dx.doi.org/10.1038/s41746-020-0256-0>.

[86] J. R.-. Imbus, R. W.-. -Randle, S. C.-. -Pitt, R. S.-. -Sippel, and D. F. -Schneider, "Machine learning to identify multigland disease in primary hyperparathyroidism," (in English), *Journal of Surgical Research,* vol. 219, pp. 173-179, 2017, doi: <http://dx.doi.org/10.1016/j.jss.2017.05.117>.

[87] Y. J. Park *et al.*, "Machine learning-based diagnosis for disseminated intravascular coagulation (DIC): Development, external validation, and comparison to scoring systems," (in English), *PLoS ONE,* vol. 13, no. 5, p. e0195861, 2018, doi: <http://dx.doi.org/10.1371/journal.pone.0195861>.

[88] Y. Zhang *et al.*, "A machine learning-based framework to identify type 2 diabetes through electronic health records," (in English), *International Journal of Medical Informatics,* vol. 97, pp. 120-127, 2017, doi: <http://dx.doi.org/10.1016/j.ijmedinf.2016.09.014>.

[89] E. Maini, B. Venkateswarlu, B. Maini, and D. Marwaha, "Machine learning-based heart disease prediction system for Indian population: An exploratory study done in South India," (in English), *Medical Journal Armed Forces India,* vol. 77, no. 3, pp. 302-311, 2021, doi: <http://dx.doi.org/10.1016/j.mjafi.2020.10.013>.

[90] V. Blanes-Selva, J. M. Garcia-Gomez, S. Tortajada, R. Vilar, and B. Valdivieso, "Machine Learning-Based Identification of Obesity from Positive and Unlabelled Electronic Health Records," (in English), *Studies in health technology and informatics,* vol. 270, pp. 864-868, 2020, doi: <http://dx.doi.org/10.3233/SHTI200284>.

[91] E. A. Jammeh *et al.*, "Machine-learning based identification of undiagnosed dementia in primary care: A feasibility study," (in English), *BJGP Open,* vol. 2, no. 2, 2018, doi: <http://dx.doi.org/10.3399/bjgpopen18X101589>.

[92] R. Mansuri *et al.*, "A multi-layer model for the early detection of COVID-19," (in English), *Journal of the Royal Society Interface,* vol. 18, no. 181, p. 20210284, 2021, doi: <http://dx.doi.org/10.1098/rsif.2021.0284>.

[93] J. F.-. Peterson *et al.*, "National Veterans Health Administration inpatient risk stratification models for hospital-acquired acute kidney injury," (in English), *Journal of the American Medical Informatics Association,* vol. 22, no. 5, pp. 1054-1071, 2015, doi: <http://dx.doi.org/10.1093/jamia/ocv051>.

[94] Y. Zhao *et al.*, "Natural language processing and machine learning for identifying incident stroke from electronic health records: Algorithm development and validation," (in English), *Journal of Medical Internet Research,* vol. 23, no. 3, p. e22951, 2021, doi: <http://dx.doi.org/10.2196/22951>.

[95] Y.-. Wang *et al.*, "NLP based congestive heart failure case finding: A prospective analysis on statewide electronic medical records," (in English), *International Journal of Medical Informatics,* vol. 84, no. 12, pp. 1039-1047, 2015, doi: <http://dx.doi.org/10.1016/j.ijmedinf.2015.06.007>.

[96] S. M. Lee *et al.*, "Nonalcoholic fatty liver disease and early prediction of gestational diabetes mellitus using machine learning methods," (in English), *Clinical and Molecular Hepatology,* vol. 28, no. 1, pp. 105-116, 2022, doi: <https://dx.doi.org/10.3350/cmh.2021.0174>.

[97] Y. X. Li *et al.*, "Novel electronic health records applied for prediction of pre-eclampsia: Machine-learning algorithms," (in English), *Pregnancy Hypertension,* vol. 26, pp. 102-109, 2021, doi: <https://dx.doi.org/10.1016/j.preghy.2021.10.006>.

[98] C.-P.-. Wu, P.-. -Zhang, J. G.-. -Zein, A. H.-. -Attaway, and A. -Nazha, "Novel Machine Learning Can Predict Acute Asthma Exacerbation," (in English), *Chest,* vol. 159, no. 5, pp. 1747-1757, 2021, doi: <http://dx.doi.org/10.1016/j.chest.2020.12.051>.

[99] C. Weber *et al.*, "Optimized identification of advanced chronic kidney disease and absence of kidney disease by combining different electronic health data resources and by applying machine learning strategies," (in English), *Journal of Clinical Medicine,* vol. 9, no. 9, pp. 1-19, 2020, doi: <http://dx.doi.org/10.3390/jcm9092955>.

[100] S.-. Le *et al.*, "Pediatric Severe Sepsis Prediction Using Machine Learning," (in English), *Frontiers in Pediatrics,* vol. 7, p. 413, 2019, doi: <http://dx.doi.org/10.3389/fped.2019.00413>.

[101] R. K. Akyea, N. Qureshi, J. Kai, and S. F. Weng, "Performance and clinical utility of supervised machine-learning approaches in detecting familial hypercholesterolaemia in primary care," (in English), *npj Digital Medicine,* vol. 3, no. 1, p. 142, 2020, doi: <http://dx.doi.org/10.1038/s41746-020-00349-5>.

[102] L. Li *et al.*, "Performance assessment of different machine learning approaches in predicting diabetic ketoacidosis in adults with type 1 diabetes using electronic health records data," (in English), *Pharmacoepidemiology and Drug Safety,* vol. 30, no. 5, pp. 610-618, 2021, doi: <http://dx.doi.org/10.1002/pds.5199>.

[103] G. R. Hart *et al.*, "Population-Based Screening for Endometrial Cancer: Human vs. Machine Intelligence," *Frontiers in artificial intelligence,* vol. 3, p. 539879, 2020, doi: <https://dx.doi.org/10.3389/frai.2020.539879>.

[104] S. J.-. Weisenthal, C.-. -Quill, S.-. -Farooq, H.-. -Kautz, and M. S. -Zand, "Predicting acute kidney injury at hospital re-entry using high-dimensional electronic health record data," *PloS one,* vol. 13, no. 11, p. e0204920, 2018, doi: <https://dx.doi.org/10.1371/journal.pone.0204920>.

[105] N. R. Hill *et al.*, "Predicting atrial fibrillation in primary care using machine learning," (in English), *PLoS ONE,* vol. 14, no. 11, p. e0224582, 2019, doi: <http://dx.doi.org/10.1371/journal.pone.0224582>.

[106] A. Akselrod-Ballin *et al.*, "Predicting breast cancer by applying deep learning to linked health records and mammograms," (in English), *Radiology,* vol. 292, no. 2, pp. 331-342, 2019, doi: <http://dx.doi.org/10.1148/radiol.2019182622>.

[107] R. Hammond *et al.*, "Predicting childhood obesity using electronic health records and publicly available data," (in English), *PLoS ONE,* vol. 14, no. 4, p. e0215571, 2019, doi: <http://dx.doi.org/10.1371/journal.pone.0215571>.

[108] K. Haas *et al.*, "Predicting dementia with routine care EMR data," (in English), *Artificial Intelligence in Medicine,* vol. 102, p. 101771, 2020, doi: <http://dx.doi.org/10.1016/j.artmed.2019.101771>.

[109] I. Segura-Bedmar, C. Colon-Ruiz, M. Moro-Moro, and M. A. Tejedor-Alonso, "Predicting of anaphylaxis in big data EMR by exploring machine learning approaches," (in English), *Journal of Biomedical Informatics,* vol. 87, pp. 50-59, 2018, doi: <http://dx.doi.org/10.1016/j.jbi.2018.09.012>.

[110] R. J. Ellis, Z. Wang, A. Ma'Ayan, and N. Genes, "Predicting opioid dependence from electronic health records with machine learning," (in English), *BioData Mining,* vol. 12, no. 1, p. 3, 2019, doi: <http://dx.doi.org/10.1186/s13040-019-0193-0>.

[111] H.-. Wu *et al.*, "Predicting post-stroke pneumonia using deep neural network approaches," (in English), *International Journal of Medical Informatics,* vol. 132, p. 103986, 2019, doi: <http://dx.doi.org/10.1016/j.ijmedinf.2019.103986>.

[112] C. S.-. Nwosu, S.-. -Dev, P.-. -Bhardwaj, B.-. -Veeravalli, and D. -John, "Predicting Stroke from Electronic Health Records," (in English), *Annual International Conference of the IEEE Engineering in Medicine and Biology Society. IEEE Engineering in Medicine and Biology Society. Annual International Conference,* vol. 2019, pp. 5704-5707, 2019, doi: <http://dx.doi.org/10.1109/EMBC.2019.8857234>.

[113] Q.-. Chen *et al.*, "Predicting suicide attempt or suicide death following a visit to psychiatric specialty care: A machine learning study using Swedish national registry data," (in English), *PLoS Medicine,* vol. 17, no. 11, p. e1003416, 2020, doi: <http://dx.doi.org/10.1371/journal.pmed.1003416>.

[114] X.-. Zhuang *et al.*, "Predicting the diagnosis of HIV and sexually transmitted infections among men who have sex with men using machine learning approaches," (in English), *Journal of Infection,* vol. 82, no. 1, pp. 48-59, 2021, doi: <http://dx.doi.org/10.1016/j.jinf.2020.11.007>.

[115] B. P. Nguyen *et al.*, "Predicting the onset of type 2 diabetes using wide and deep learning with electronic health records," (in English), *Computer Methods and Programs in Biomedicine,* vol. 182, p. 105055, 2019, doi: <http://dx.doi.org/10.1016/j.cmpb.2019.105055>.

[116] R. A.-. Taylor, C. L.-. -Moore, K.-H.-. -Cheung, and C. -Brandt, "Predicting urinary tract infections in the emergency department with machine learning," (in English), *PLoS ONE,* vol. 13, no. 3, p. e0194085, 2018, doi: <http://dx.doi.org/10.1371/journal.pone.0194085>.

[117] L.-. He *et al.*, "Predicting venous thromboembolism in hospitalized trauma patients: a combination of the Caprini score and data-driven machine learning model," (in English), *BMC Emergency Medicine,* vol. 21, no. 1, p. 60, 2021, doi: <http://dx.doi.org/10.1186/s12873-021-00447-x>.

[118] H.-. Mohamadlou *et al.*, "Prediction of Acute Kidney Injury With a Machine Learning Algorithm Using Electronic Health Record Data," (in English), *Canadian Journal of Kidney Health and Disease,* vol. 5, 2018, doi: <http://dx.doi.org/10.1177/2054358118776326>.

[119] J. Fan *et al.*, "The prediction of asymptomatic carotid atherosclerosis with electronic health records: a comparative study of six machine learning models," (in English), *BMC medical informatics and decision making,* vol. 21, no. 1, p. 115, 2021, doi: <http://dx.doi.org/10.1186/s12911-021-01480-3>.

[120] K. H. Lee *et al.*, "Prediction of Bacteremia Based on 12-Year Medical Data Using a Machine Learning Approach: Effect of Medical Data by Extraction Time," (in English), *Diagnostics,* vol. 12, no. 1, p. 102, 2022, doi: <https://dx.doi.org/10.3390/diagnostics12010102>.

[121] A. Allen *et al.*, "Prediction of diabetic kidney disease with machine learning algorithms, upon the initial diagnosis of type 2 diabetes mellitus," (in English), *BMJ Open Diabetes Research and Care,* vol. 10, no. 1, p. e002560, 2022, doi: <https://dx.doi.org/10.1136/bmjdrc-2021-002560>.

[122] W. Wang *et al.*, "Prediction of Endometrial Carcinoma Using the Combination of Electronic Health Records and an Ensemble Machine Learning Method," (in English), *Frontiers in Medicine,* vol. 9, p. 851890, 2022, doi: <https://dx.doi.org/10.3389/fmed.2022.851890>.

[123] N. S. Artzi *et al.*, "Prediction of gestational diabetes based on nationwide electronic health records," (in English), *Nature Medicine,* vol. 26, no. 1, pp. 71-76, 2020, doi: <http://dx.doi.org/10.1038/s41591-019-0724-8>.

[124] D. Mandair, P. Tiwari, S. Simon, M. A. Rosenberg, and K. L. Colborn, "Prediction of incident myocardial infarction using machine learning applied to harmonized electronic health record data," (in English), *BMC medical informatics and decision making,* vol. 20, no. 1, p. 252, 2020, doi: <http://dx.doi.org/10.1186/s12911-020-01268-x>.

[125] H.-. Lin *et al.*, "Prediction of myopia development among Chinese school-aged children using refraction data from electronic medical records: A retrospective, multicentre machine learning study," (in English), *PLoS Medicine,* vol. 15, no. 11, p. e1002674, 2018, doi: <http://dx.doi.org/10.1371/journal.pmed.1002674>.

[126] G.-. Tang *et al.*, "Prediction of Sepsis in COVID-19 Using Laboratory Indicators," (in English), *Frontiers in Cellular and Infection Microbiology,* vol. 10, p. 586054, 2020, doi: <http://dx.doi.org/10.3389/fcimb.2020.586054>.

[127] P.-. Fan *et al.*, "Prediction of suicide-related events by analyzing electronic medical records from PTSD patients with bipolar disorder," (in English), *Brain Sciences,* vol. 10, no. 11, pp. 1-30, 2020, doi: <http://dx.doi.org/10.3390/brainsci10110784>.

[128] X.-. Wang *et al.*, "Prediction of the 1-Year Risk of Incident Lung Cancer: Prospective Study Using Electronic Health Records from the State of Maine," (in English), *Journal of medical Internet research,* vol. 21, no. 5, p. e13260, 2019, doi: <http://dx.doi.org/10.2196/13260>.

[129] O. Houri *et al.*, "Prediction of Type 2 Diabetes Mellitus According to Glucose Metabolism Patterns in Pregnancy Using a Novel Machine Learning Algorithm," (in English), *Journal of Medical and Biological Engineering,* vol. 42, no. 1, pp. 138-144, 2022, doi: <https://dx.doi.org/10.1007/s40846-022-00685-9>.

[130] A. P.-. Cox, M.-. -Raluy-Callado, M.-. -Wang, A. M.-. -Bakheit, A. P.-. -Moore, and J. -Dinet, "Predictive analysis for identifying potentially undiagnosed post-stroke spasticity patients in United Kingdom," (in English), *Journal of Biomedical Informatics,* vol. 60, pp. 328-333, 2016, doi: <http://dx.doi.org/10.1016/j.jbi.2016.02.012>.

[131] W. Li *et al.*, "Predictive model and risk analysis for diabetic retinopathy using machine learning: A retrospective cohort study in China," (in English), *BMJ Open,* vol. 11, no. 11, p. e050989, 2021, doi: <https://dx.doi.org/10.1136/bmjopen-2021-050989>.

[132] W.-. Song, S. Y.-. -Jung, H.-. -Baek, C. W.-. -Choi, Y. H.-. -Jung, and S. -Yoo, "A Predictive Model Based on Machine Learning for the Early Detection of Late-Onset Neonatal Sepsis: Development and Observational Study," *JMIR medical informatics,* vol. 8, no. 7, p. e15965, 2020, doi: <https://dx.doi.org/10.2196/15965>.

[133] R. Kop *et al.*, "Predictive modeling of colorectal cancer using a dedicated pre-processing pipeline on routine electronic medical records," (in English), *Computers in Biology and Medicine,* vol. 76, pp. 30-38, 2016, doi: <http://dx.doi.org/10.1016/j.compbiomed.2016.06.019>.

[134] R. Huang, M. D. Nemesure, M. V. Heinz, and N. C. Jacobson, "Predictive modeling of depression and anxiety using electronic health records and a novel machine learning approach with artificial intelligence," (in English), *Scientific reports,* vol. 11, no. 1, p. 1980, 2021, doi: <http://dx.doi.org/10.1038/s41598-021-81368-4>.

[135] G.-. Yu *et al.*, "The role of artificial intelligence in identifying asthma in pediatric inpatient setting," (in English), *Annals of Translational Medicine,* vol. 8, no. 21, p. 1367, 2020, doi: <http://dx.doi.org/10.21037/ATM-20-2501A>.

[136] L. Jamian, L. Wheless, L. J. Crofford, and A. Barnado, "Rule-based and machine learning algorithms identify patients with systemic sclerosis accurately in the electronic health record," (in English), *Arthritis Research and Therapy,* vol. 21, no. 1, p. 305, 2019, doi: <http://dx.doi.org/10.1186/s13075-019-2092-7>.

[137] A. Pimentel, H. Gamboa, A. V. Carreiro, and R. T. Ribeiro, "Screening diabetes mellitus 2 based on electronic health records using temporal features," (in English), *Health informatics journal,* vol. 24, no. 2, pp. 194-205, 2018, doi: <http://dx.doi.org/10.1177/1460458216663023>.

[138] Y.-. Ni *et al.*, "Towards phenotyping stroke: Leveraging data from a large-scale epidemiological study to detect stroke diagnosis," (in English), *PLoS ONE,* vol. 13, no. 2, p. e0192586, 2018, doi: <http://dx.doi.org/10.1371/journal.pone.0192586>.

[139] S. Mani, Y. Chen, T. Elasy, W. Clayton, and J. Denny, "Type 2 diabetes risk forecasting from EMR data using machine learning," (in English), *AMIA ... Annual Symposium proceedings / AMIA Symposium. AMIA Symposium,* vol. 2012, pp. 606-615, 2012. [Online]. Available: <http://ovidsp.ovid.com/ovidweb.cgi?T=JS&PAGE=reference&D=emed13&NEWS=N&AN=369427527//https://www.ncbi.nlm.nih.gov/pmc/articles/PMC3540444/pdf/amia_2012_symp_0606.pdf>.

[140] J. Pathak, Y. Zhang, and S. Wang, "Using Electronic Health Records and Machine Learning to Predict Postpartum Depression," (in English), *Studies in health technology and informatics,* vol. 264, pp. 888-892, 2019, doi: <http://dx.doi.org/10.3233/SHTI190351>.

[141] S.-. DuBrava *et al.*, "Using Random Forest Models to Identify Correlates of a Diabetic Peripheral Neuropathy Diagnosis from Electronic Health Record Data," (in English), *Pain medicine (Malden, Mass.),* vol. 18, no. 1, pp. 107-115, 2017, doi: <http://dx.doi.org/10.1093/pm/pnw096>.

[142] H. Hussan *et al.*, "Utility of machine learning in developing a predictive model for early-age-onset colorectal neoplasia using electronic health records," (in English), *PLoS ONE,* vol. 17, no. 3 March, p. e0265209, 2022, doi: <https://dx.doi.org/10.1371/journal.pone.0265209>.

[143] Y.-. Zhou, Y.-. -Wang, Y.-. -Wei, H.-. -Yang, Q.-. -Wu, and J. -Li, "Utilizing imbalanced electronic health records to predict acute kidney injury by ensemble learning and time series model," (in English), *BMC medical informatics and decision making,* vol. 20, no. 1, p. 238, 2020, doi: <http://dx.doi.org/10.1186/s12911-020-01245-4>.

[144] J. L.-. Schneider, E.-. -Layefsky, N.-. -Udaltsova, T. R.-. -Levin, and D. A. -Corley, "Validation of an Algorithm to Identify Patients at Risk for Colorectal Cancer Based on Laboratory Test and Demographic Data in Diverse, Community-Based Population," (in English), *Clinical Gastroenterology and Hepatology,* vol. 18, no. 12, p. 2734, 2020, doi: <http://dx.doi.org/10.1016/j.cgh.2020.04.054>.

[145] C. A.-. Turner *et al.*, "Word2Vec inversion and traditional text classifiers for phenotyping lupus," (in English), *BMC medical informatics and decision making,* vol. 17, no. 1, p. 126, 2017, doi: <http://dx.doi.org/10.1186/s12911-017-0518-1>.
