# Supplementary material for: Ensemble machine learning methods in screening electronic health records: A scoping review
Source: Digit Health. 2023 May 9;9:20552076231173225. doi: 10.1177/20552076231173225 (PMC10176785; doi:10.1177/20552076231173225)
Supplement: sj-docx-6-dhj-10.1177_20552076231173225 - Supplemental material for Ensemble machine learning methods in screening electronic health records: A scoping review [file sj-docx-6-dhj-10.1177_20552076231173225.docx]

**Supplemental Reference 2: Articles reporting on ML models for medical screening of EHRs but without EML, excluded from the analysis.**

[1-79]

[1] K. G. Pollock *et al.*, "Application of a machine learning algorithm for detection of atrial fibrillation in secondary care," (in English), *IJC Heart and Vasculature,* vol. 31, p. 100674, 2020, doi: <http://dx.doi.org/10.1016/j.ijcha.2020.100674>.

[2] A. Guo, S. Smith, Y. M. Khan, J. R. Langabeer, and R. E. Foraker, "Application of a time-series deep learning model to predict cardiac dysrhythmias in electronic health records," (in English), *PLoS ONE,* vol. 16, no. 9 September, p. e0239007, 2021, doi: <http://dx.doi.org/10.1371/journal.pone.0239007>.

[3] P. Wilansky, V. de Luca, A. Roy, J. L. Kennedy, J. Geraci, and J. Strauss, "Applying deep neural networks to unstructured text notes in electronic medical records for phenotyping youth depression," (in English), *Evidence-based mental health,* vol. 20, no. 3, pp. 83-87, 2017, doi: <http://dx.doi.org/10.1136/eb-2017-102688>.

[4] N. K. Tran *et al.*, "Artificial intelligence and machine learning for predicting acute kidney injury in severely burned patients: A proof of concept," (in English), *Burns,* vol. 45, no. 6, pp. 1350-1358, 2019, doi: <http://dx.doi.org/10.1016/j.burns.2019.03.021>.

[5] G. N. Ioannou *et al.*, "Assessment of a Deep Learning Model to Predict Hepatocellular Carcinoma in Patients with Hepatitis C Cirrhosis," (in English), *JAMA Network Open,* 2020, doi: <http://dx.doi.org/10.1001/jamanetworkopen.2020.15626>.

[6] C.-W. Liang, H.-H. Wang, Y.-H. Wang, and Y.-C. Li, "Assessment of Deep Learning Using Nonimaging Information and Sequential Medical Records to Develop a Prediction Model for Nonmelanoma Skin Cancer," (in English), *JAMA Dermatology,* vol. 155, no. 11, pp. 1277-1283, 2019, doi: <http://dx.doi.org/10.1001/jamadermatol.2019.2335>.

[7] A. Bachtel, Y. Ni, K. Nause, and S. Beal, "Automated detection of substance use information from electronic health records for a pediatric population," (in English), *Journal of the American Medical Informatics Association : JAMIA,* 2021, doi: <http://dx.doi.org/10.1093/jamia/ocab116>.

[8] Z. Afzal *et al.*, "Automatic generation of case-detection algorithms to identify children with asthma from large electronic health record databases," (in English), *Pharmacoepidemiology and Drug Safety,* vol. 22, no. 8, pp. 826-833, 2013, doi: <http://dx.doi.org/10.1002/pds.3438>.

[9] C. Lin *et al.*, "Automatic Prediction of Rheumatoid Arthritis Disease Activity from the Electronic Medical Records," (in English), *PLoS ONE,* vol. 8, no. 8, p. e69932, 2013, doi: <http://dx.doi.org/10.1371/journal.pone.0069932>.

[10] B. N. Patel and C. P. Langlotz, "Beyond the AJR: deep learning using chest radiographs to identify high-risk smokers for lung cancer screening computed tomography: Development and validation of a prediction model"," (in English), *American Journal of Roentgenology,* vol. 217, no. 2, p. 521, 2021, doi: <https://dx.doi.org/10.2214/AJR.20.25334>.

[11] Y. Meng, W. Speier, M. K. Ong, and C. W. Arnold, "Bidirectional Representation Learning from Transformers Using Multimodal Electronic Health Record Data to Predict Depression," (in English), *IEEE Journal of Biomedical and Health Informatics,* vol. 25, no. 8, pp. 3121-3129, 2021, doi: <http://dx.doi.org/10.1109/JBHI.2021.3063721>.

[12] S. K. Tedeschi *et al.*, "Classifying Pseudogout Using Machine Learning Approaches With Electronic Health Record Data," (in English), *Arthritis Care and Research,* vol. 73, no. 3, pp. 442-448, 2021, doi: <http://dx.doi.org/10.1002/acr.24132>.

[13] A. Gupta, T. Liu, and S. Shepherd, "Clinical decision support system to assess the risk of sepsis using Tree Augmented Bayesian networks and electronic medical record data," (in English), *Health informatics journal,* vol. 26, no. 2, pp. 841-861, 2020, doi: <http://dx.doi.org/10.1177/1460458219852872>.

[14] B. Ustun, M. B. Westover, C. Rudin, and M. T. Bianchi, "Clinical prediction models for sleep apnea: The importance of medical history over symptoms," (in English), *Journal of Clinical Sleep Medicine,* vol. 12, no. 2, pp. 161-168, 2016, doi: <http://dx.doi.org/10.5664/jcsm.5476>.

[15] D. Tjandra, J. Wiens, R. Q. Migrino, and B. Giordani, "Cohort discovery and risk stratification for Alzheimer's disease: an electronic health record-based approach," (in English), *Alzheimer's and Dementia: Translational Research and Clinical Interventions,* vol. 6, no. 1, p. e12035, 2020, doi: <http://dx.doi.org/10.1002/trc2.12035>.

[16] H. Park *et al.*, "Comparison of approaches for heart failure case identification from electronic health record data," (in English), *JAMA Cardiology,* vol. 1, no. 9, pp. 1014-1020, 2016, doi: <http://dx.doi.org/10.1001/jamacardio.2016.3236>.

[17] J. Agbeve, E. S. Owusu Adjah, O. Montvida, and S. K. Paul, "Data mining approach to identify disease cohorts from primary care electronic medical records: A case of diabetes mellitus," (in English), *Open Bioinformatics Journal,* vol. 10, pp. 16-27, 2017, doi: <http://dx.doi.org/10.2174/1875036201710010016>.

[18] C. S. Lee, D. M. Baughman, and A. Y. Lee, "Deep learning is effective for the classification of OCT images of normal versus Age-related Macular Degeneration," (in English), *Ophthalmology. Retina,* vol. 1, no. 4, pp. 322-327, 2017, doi: <http://dx.doi.org/10.1016/j.oret.2016.12.009>.

[19] A. Tabaie, E. W. Orenstein, S. Nemati, R. K. Basu, G. D. Clifford, and R. Kamaleswaran, "Deep Learning Model to Predict Serious Infection Among Children With Central Venous Lines," *Frontiers in pediatrics,* vol. 9, p. 726870, 2021, doi: <https://dx.doi.org/10.3389/fped.2021.726870>.

[20] I. Peis *et al.*, "Deep Sequential Models for Suicidal Ideation from Multiple Source Data," (in English), *IEEE Journal of Biomedical and Health Informatics,* vol. 23, no. 6, pp. 2286-2293, 2019, doi: <http://dx.doi.org/10.1109/JBHI.2019.2919270>.

[21] S.-M. F.-G. Zhou, Fabiola//Kennedy, Jonathan//Cooksey, Roxanne//Siebert, Stefan//Dixon, William G.//Choy, Ernest//Danesh, John//Brayne, Carol//Allen, Naomi//Collins, Rory//Landray, Martin//Sprosen, Tim//Atkinson, Mark//Brophy, Sinead//Lyons, Ronan//Blaveri, Ekaterini//Brannan, Rachael//Denaxas, Spiros//Chaturvedi, Nish//Eastwood, Sophie//Hemingway, Harry//DeLusignan, Simon//Desai, Parul//Gallacher, John//Hotopf, Matthew//Moller, Henrik//McGilchrist, Mark//Sullivan, Frank//O'Neill, Terence W.//Pringle, Mike//Strachan, David//Sudlow, Cathie//Zhang, Qiuli//Flaig, Robin//Woodfield, Rebecca, "Defining disease phenotypes in primary care electronic health records by a machine learning approach: A case study in identifying rheumatoid arthritis," (in English), *PLoS ONE,* vol. 11, no. 5, p. e0154515, 2016, doi: <http://dx.doi.org/10.1371/journal.pone.0154515>.

[22] J. Zhao *et al.*, "Detecting time-evolving phenotypic topics via tensor factorization on electronic health records: Cardiovascular disease case study," (in English), *Journal of Biomedical Informatics,* vol. 98, p. 103270, 2019, doi: <http://dx.doi.org/10.1016/j.jbi.2019.103270>.

[23] S. Sekelj *et al.*, "Detecting undiagnosed atrial fibrillation in UK primary care: Validation of a machine learning prediction algorithm in a retrospective cohort study," (in English), *European Journal of Preventive Cardiology,* 2020, doi: <http://dx.doi.org/10.1177/2047487320942338>.

[24] Y. Shao, Q. T. Zeng, K. K. Chen, S. M. Thielke, A. Shutes-David, and D. W. Tsuang, "Detection of probable dementia cases in undiagnosed patients using structured and unstructured electronic health records," (in English), *BMC medical informatics and decision making,* vol. 19, no. 1, p. 128, 2019, doi: <http://dx.doi.org/10.1186/s12911-019-0846-4>.

[25] B. C. Lethebe *et al.*, "Developing a case definition for type 1 diabetes mellitus in a primary care electronic medical record database: an exploratory study," *CMAJ open,* vol. 7, no. 2, pp. E246-E251, 2019, doi: <https://dx.doi.org/10.9778/cmajo.20180142>.

[26] T. Lingren *et al.*, "Developing an Algorithm to Detect Early Childhood Obesity in Two Tertiary Pediatric Medical Centers," (in English), *Applied clinical informatics,* vol. 7, no. 3, pp. 693-706, 2016, doi: <http://dx.doi.org/10.4338/ACI-2016-01-RA-0015>.

[27] L. Wilkinson, N. Yi, S. Judd, T. Mehta, and W. Timothy Garvey, "Development and validation of a model for predicting incident type 2 diabetes using quantitative clinical data and a Bayesian logistic model: A nationwide cohort and modeling study," (in English), *PLoS Medicine,* vol. 17, no. 8, p. e1003232, 2020, doi: <http://dx.doi.org/10.1371/JOURNAL.PMED.1003232>.

[28] C. Brusq, R. Mieusset, and S. M. Hamdi, "Development of a multivariable prediction model for congenital unilateral absence of the vas deferens in male partners of infertile couples," (in English), *Andrology,* 2021, doi: <http://dx.doi.org/10.1111/andr.13106>.

[29] C. J. Danford, M. Lai, J. Y. Lee, I. A. Strohbehn, and K. E. Corey, "Development of an Algorithm to Identify Cases of Nonalcoholic Steatohepatitis Cirrhosis in the Electronic Health Record," (in English), *Digestive Diseases and Sciences,* vol. 66, no. 5, pp. 1452-1460, 2021, doi: <http://dx.doi.org/10.1007/s10620-020-06388-y>.

[30] J. Baker *et al.*, "Development, validation, and proof-of-concept implementation of a two-year risk prediction model for undiagnosed atrial fibrillation using common electronic health data (UNAFIED)," (in English), *BMC medical informatics and decision making,* vol. 21, no. 1, p. 112, 2021, doi: <http://dx.doi.org/10.1186/s12911-021-01482-1>.

[31] H. Park *et al.*, "Early Identification of Patients With Acute Decompensated Heart Failure," (in English), *Journal of Cardiac Failure,* vol. 24, no. 6, pp. 357-362, 2018, doi: <http://dx.doi.org/10.1016/j.cardfail.2017.08.458>.

[32] D. Hong, L. Shi, D. Fort, and E. G. Price-Haywood, "Electronic Medical Record Risk Modeling of Cardiovascular Outcomes Among Patients with Type 2 Diabetes," (in English), *Diabetes Therapy,* vol. 12, no. 7, pp. 2007-2017, 2021, doi: <http://dx.doi.org/10.1007/s13300-021-01096-w>.

[33] S. A. Weston, C. G. Chute, R. Meverden, S. J. Jacobsen, V. L. Roger, and S. Pakhomov, "Electronic medical records for clinical research: Application to the identification of heart failure," (in English), *American Journal of Managed Care,* vol. 13, no. 6 I, pp. 281-288, 2007. [Online]. Available: <http://ovidsp.ovid.com/ovidweb.cgi?T=JS&PAGE=reference&D=emed10&NEWS=N&AN=46983137>.

[34] T. E. Lloyd, A. L. Mammen, A. A. Amato, M. D. Weiss, M. Needham, and S. A. Greenberg, "Evaluation and construction of diagnostic criteria for inclusion body myositis," (in English), *Neurology,* vol. 83, no. 5, pp. 426-433, 2014, doi: <http://dx.doi.org/10.1212/WNL.0000000000000642>.

[35] Y. Lin *et al.*, "External validation of a machine learning classifier to identify unhealthy alcohol use in hospitalized patients," (in English), *Addiction (Abingdon, England),* vol. 117, no. 4, pp. 925-933, 2022, doi: <https://dx.doi.org/10.1111/add.15730>.

[36] R. L. Figueroa and C. A. Flores, "Extracting Information from Electronic Medical Records to Identify the Obesity Status of a Patient Based on Comorbidities and Bodyweight Measures," (in English), *Journal of Medical Systems,* vol. 40, no. 8, p. 191, 2016, doi: <http://dx.doi.org/10.1007/s10916-016-0548-8>.

[37] R. Weegar, H. Dalianis, M. Kvist, K. Sundstrom, and S. Brunak, "Finding Cervical Cancer Symptoms in Swedish Clinical Text using a Machine Learning Approach and NegEx," (in English), *AMIA ... Annual Symposium proceedings. AMIA Symposium,* vol. 2015, pp. 1296-1305, 2015. [Online]. Available: <http://ovidsp.ovid.com/ovidweb.cgi?T=JS&PAGE=reference&D=emed16&NEWS=N&AN=621604620-//-https://www.ncbi.nlm.nih.gov/pmc/articles/PMC4765575/pdf/2245432.pdf>.

[38] H. R. Chua *et al.*, "Health Care Analytics with Time-Invariant and Time-Variant Feature Importance to Predict Hospital-Acquired Acute Kidney Injury: Observational Longitudinal Study," (in English), *Journal of Medical Internet Research,* vol. 23, no. 12, p. e30805, 2021, doi: <https://dx.doi.org/10.2196/30805>.

[39] W.-Q. Wei, C. Tao, G. Jiang, and C. G. Chute, "A high throughput semantic concept frequency based approach for patient identification: a case study using type 2 diabetes mellitus clinical notes," (in English), *AMIA ... Annual Symposium proceedings / AMIA Symposium. AMIA Symposium,* vol. 2010, pp. 857-861, 2010. [Online]. Available: <http://ovidsp.ovid.com/ovidweb.cgi?T=JS&PAGE=reference&D=emed11&NEWS=N&AN=611776371-//-https://www.ncbi.nlm.nih.gov/pmc/articles/PMC3041302/pdf/amia-2010_sympproc_0857.pdf>.

[40] S.-T. Liaw, P. Ray, J. Jonnagaddala, M. Kumar, H.-J. Dai, and C.-Y. Hsu, "Identification and Progression of Heart Disease Risk Factors in Diabetic Patients from Longitudinal Electronic Health Records," (in English), *BioMed Research International,* vol. 2015, p. 636371, 2015, doi: <http://dx.doi.org/10.1155/2015/636371>.

[41] N. R. Hill *et al.*, "Identification of undiagnosed atrial fibrillation patients using a machine learning risk prediction algorithm and diagnostic testing (PULsE-AI): Study protocol for a randomised controlled trial," (in English), *Contemporary Clinical Trials,* vol. 99, p. 106191, 2020, doi: <http://dx.doi.org/10.1016/j.cct.2020.106191>.

[42] A. Jorge *et al.*, "Identifying lupus patients in electronic health records: Development and validation of machine learning algorithms and application of rule-based algorithms," (in English), *Seminars in Arthritis and Rheumatism,* vol. 49, no. 1, pp. 84-90, 2019, doi: <http://dx.doi.org/10.1016/j.semarthrit.2019.01.002>.

[43] L. Zhou *et al.*, "Identifying Patients with Depression Using Free-text Clinical Documents," (in English), *Studies in health technology and informatics,* vol. 216, pp. 629-633, 2015. [Online]. Available: <http://ovidsp.ovid.com/ovidweb.cgi?T=JS&PAGE=reference&D=emed16&NEWS=N&AN=615155210-//-https://ebooks.iospress.nl/pdf/doi/10.3233/978-1-61499-564-7-629>.

[44] A. Talaei-Khoei and J. M. Wilson, "Identifying people at risk of developing type 2 diabetes: A comparison of predictive analytics techniques and predictor variables," (in English), *International Journal of Medical Informatics,* vol. 119, pp. 22-38, 2018, doi: <http://dx.doi.org/10.1016/j.ijmedinf.2018.08.008>.

[45] F. Ge, Y. Wang, C. Yuan, W. Zhang, and J. Jiang, "Identifying suicidal ideation among chinese patients with major depressive disorder: Evidence from a real-world hospital-based study in China," (in English), *Neuropsychiatric Disease and Treatment,* vol. 16, pp. 665-672, 2020, doi: <http://dx.doi.org/10.2147/NDT.S238286>.

[46] S. L. Sheth, Paul//Bajaj, Archna//Cuchel, Marina//Hajj, Jihane//Soffer, Daniel E.//Webb, Gayley//Risman, Marjorie//Rader, Daniel J.//Jacoby, Douglas//Hossain, Erik//Borovskiy, Yulia//Myers, Kelly D.//Wilemon, Katherine A., "Implementation of a Machine-Learning Algorithm in the Electronic Health Record for Targeted Screening for Familial Hypercholesterolemia: A Quality Improvement Study," (in English), *Circulation: Cardiovascular Quality and Outcomes,* pp. 746-750, 2021, doi: <http://dx.doi.org/10.1161/CIRCOUTCOMES.120.007641>.

[47] Z. Afzal, M. J. Schuemie, J. C. van Blijderveen, E. F. Sen, M. C. J. M. Sturkenboom, and J. A. Kors, "Improving sensitivity of machine learning methods for automated case identification from free-text electronic medical records," (in English), *BMC medical informatics and decision making,* vol. 13, p. 30, 2013, doi: <http://dx.doi.org/10.1186/1472-6947-13-30>.

[48] N. J. Goodson *et al.*, "Incorporating natural language processing to improve classification of axial spondyloarthritis using electronic health records," (in English), *Rheumatology (United Kingdom),* vol. 59, no. 5, pp. 1059-1065, 2020, doi: <http://dx.doi.org/10.1093/rheumatology/kez375>.

[49] H. Choo, M. Kim, J. Choi, J. Shin, and S.-Y. Shin, "Influenza Screening via Deep Learning Using a Combination of Epidemiological and Patient-Generated Health Data: Development and Validation Study," (in English), *Journal of Medical Internet Research,* vol. 22, no. 10, p. e21369, 2020, doi: <http://dx.doi.org/10.2196/21369>.

[50] H. Bai *et al.*, "Integrating exosomal microRNAs and electronic health data improved tuberculosis diagnosis," (in English), *EBioMedicine,* vol. 40, pp. 564-573, 2019, doi: <http://dx.doi.org/10.1016/j.ebiom.2019.01.023>.

[51] E. Gustafson, J. Pacheco, F. Wehbe, J. Silverberg, and W. Thompson, "A Machine Learning Algorithm for Identifying Atopic Dermatitis in Adults from Electronic Health Records," *IEEE International Conference on Healthcare Informatics. IEEE International Conference on Healthcare Informatics,* vol. 2017, pp. 83-90, 2017, doi: <https://dx.doi.org/10.1109/ICHI.2017.31>.

[52] C. Su, F. Wang, R. Aseltine, R. Doshi, K. Chen, and S. C. Rogers, "Machine learning for suicide risk prediction in children and adolescents with electronic health records," (in English), *Translational Psychiatry,* vol. 10, no. 1, p. 413, 2020, doi: <http://dx.doi.org/10.1038/s41398-020-01100-0>.

[53] G. A. Klados *et al.*, "Machine Learning Model for Predicting CVD Risk on NHANES Data," (in English), *Annual International Conference of the IEEE Engineering in Medicine and Biology Society. IEEE Engineering in Medicine and Biology Society. Annual International Conference,* vol. 2021, pp. 1749-1752, 2021, doi: <https://dx.doi.org/10.1109/EMBC46164.2021.9630119>.

[54] C.-N. L. Hsu, Chien-Liang//Kuo, Chin-Yu//Lin, Yun-Chun//Tain, You-Lin, "Machine learning model for risk prediction of community-acquired acute kidney injury hospitalization from electronic health records: Development and validation study," (in English), *Journal of Medical Internet Research,* vol. 22, no. 8, p. e16903, 2020, doi: <http://dx.doi.org/10.2196/16903>.

[55] I. Persson, A. Ostling, M. Arlbrandt, J. Soderberg, and D. Becedas, "A Machine Learning Sepsis Prediction Algorithm for Intended Intensive Care Unit Use (NAVOY Sepsis): Proof-of-Concept Study," *JMIR formative research,* vol. 5, no. 9, p. e28000, 2021, doi: <https://dx.doi.org/10.2196/28000>.

[56] N. Shang *et al.*, "Medical records-based chronic kidney disease phenotype for clinical care and "big data" observational and genetic studies," (in English), *npj Digital Medicine,* vol. 4, no. 1, p. 70, 2021, doi: <http://dx.doi.org/10.1038/s41746-021-00428-1>.

[57] D. To *et al.*, "Natural language processing and machine learning to identify alcohol misuse from the electronic health record in trauma patients: development and internal validation," (in English), *Journal of the American Medical Informatics Association,* vol. 26, no. 3, pp. 254-261, 2019, doi: <http://dx.doi.org/10.1093/jamia/ocy166>.

[58] C. Leonard Westgate, M. Levis, J. Gui, B. V. Watts, and B. Shiner, "Natural language processing of clinical mental health notes may add predictive value to existing suicide risk models," (in English), *Psychological medicine,* pp. 1-10, 2020, doi: <http://dx.doi.org/10.1017/S0033291720000173>.

[59] L. C. McKernan, M. C. Lenert, L. J. Crofford, and C. G. Walsh, "Outpatient Engagement and Predicted Risk of Suicide Attempts in Fibromyalgia," (in English), *Arthritis Care and Research,* vol. 71, no. 9, pp. 1255-1263, 2019, doi: <http://dx.doi.org/10.1002/acr.23748>.

[60] Q. Yuan *et al.*, "Performance of a Machine Learning Algorithm Using Electronic Health Record Data to Identify and Estimate Survival in a Longitudinal Cohort of Patients with Lung Cancer," (in English), *JAMA Network Open,* vol. 4, no. 7, p. e2114723, 2021, doi: <http://dx.doi.org/10.1001/jamanetworkopen.2021.14723>.

[61] J. Honerlaw *et al.*, "A phenotyping algorithm to identify acute ischemic stroke accurately from a national biobank: The million veteran program," (in English), *Clinical Epidemiology,* vol. 10, pp. 1509-1521, 2018, doi: <http://dx.doi.org/10.2147/CLEP.S160764>.

[62] S. H. Oh, S. J. Lee, and J. Park, "Precision Medicine for Hypertension Patients with Type 2 Diabetes via Reinforcement Learning," (in English), *Journal of Personalized Medicine,* vol. 12, no. 1, p. 87, 2022, doi: <https://dx.doi.org/10.3390/jpm12010087>.

[63] H. Jin, S. Wu, I. Vidyanti, P. Di Capua, and B. Wu, "Predicting Depression among Patients with Diabetes Using Longitudinal Data. A Multilevel Regression Model," (in English), *Methods of information in medicine,* vol. 54, no. 6, pp. 553-559, 2015, doi: <http://dx.doi.org/10.3414/ME14-02-0009>.

[64] J. B. Edgcomb, T. Shaddox, J. O. Brooks, and G. Hellemann, "Predicting suicidal behavior and self-harm after general hospitalization of adults with serious mental illness," (in English), *Journal of Psychiatric Research,* vol. 136, pp. 515-521, 2021, doi: <http://dx.doi.org/10.1016/j.jpsychires.2020.10.024>.

[65] M. C. Shedden-Mora, B. Lowe, and P. Jordan, "Predicting suicidal ideation in primary care: An approach to identify easily assessable key variables," (in English), *General Hospital Psychiatry,* vol. 51, pp. 106-111, 2018, doi: <http://dx.doi.org/10.1016/j.genhosppsych.2018.02.002>.

[66] R. B. Penfold *et al.*, "Predicting suicide attempts and suicide deaths among adolescents following outpatient visits," (in English), *Journal of Affective Disorders,* vol. 294, pp. 39-47, 2021, doi: <http://dx.doi.org/10.1016/j.jad.2021.06.057>.

[67] Z. Hussain, S. A. Shah, M. Mukherjee, and A. Sheikh, "Predicting the risk of asthma attacks in children, adolescents and adults: Protocol for a machine learning algorithm derived from a primary care-based retrospective cohort," (in English), *BMJ Open,* vol. 10, no. 7, p. e036099, 2020, doi: <http://dx.doi.org/10.1136/bmjopen-2019-036099>.

[68] B. Oh, J.-Y. Yun, E. C. Yeo, D.-H. Kim, J. Kim, and B.-J. Cho, "Prediction of suicidal ideation among korean adults using machine learning: A cross-sectional study," (in English), *Psychiatry Investigation,* vol. 17, no. 4, pp. 331-340, 2020, doi: <http://dx.doi.org/10.30773/pi.2019.0270>.

[69] E. Garcia-Garcia, G. M. Gonzalez-Romero, G. Escobar-Aguilar, E. M. Martin-Perez, E. d. D. Zapata Cornejo, and M. F. Cardenas Bonnet, "Real-world data and machine learning to predict cardiac amyloidosis," (in English), *International Journal of Environmental Research and Public Health,* vol. 18, no. 3, pp. 1-15, 2021, doi: <http://dx.doi.org/10.3390/ijerph18030908>.

[70] C.-C. Shih, C.-J. Lu, G.-D. Chen, and C.-C. Chang, "Risk prediction for early chronic kidney disease: Results from an adult health examination program of 19,270 individuals," (in English), *International Journal of Environmental Research and Public Health,* vol. 17, no. 14, pp. 1-11, 2020, doi: <http://dx.doi.org/10.3390/ijerph17144973>.

[71] S. Ding, H. Huang, Z. Li, S. Yang, and X. Liu, "SCNET: A Novel UGI Cancer Screening Framework Based on Semantic-Level Multimodal Data Fusion," (in English), *IEEE Journal of Biomedical and Health Informatics,* vol. 25, no. 1, pp. 143-151, 2021, doi: <http://dx.doi.org/10.1109/JBHI.2020.2983126>.

[72] T. H. McCoy, A. M. Pellegrini, R. H. Perlis, L. Han, R. E. Tanzi, and S. Berretta, "Stratifying risk for dementia onset using large-scale electronic health record data: A retrospective cohort study," (in English), *Alzheimer's and Dementia,* vol. 16, no. 3, pp. 531-540, 2020, doi: <http://dx.doi.org/10.1016/j.jalz.2019.09.084>.

[73] E. H. Weissler, J. M. Zhang, S. P. Lippmann, W. S. Jones, S. M. Rusincovitch, and R. P. Henao, "Use of natural language processing to improve identification of patients with peripheral artery disease," (in English), *Circulation: Cardiovascular Interventions,* p. e009447, 2020, doi: <http://dx.doi.org/10.1161/CIRCINTERVENTIONS.120.009447>.

[74] B. Y. Li, J. Oh, J. Wiens, K. Rao, and V. B. Young, "Using Machine learning and the electronic health record to predict complicated clostridium difficile infection," (in English), *Open Forum Infectious Diseases,* vol. 6, no. 5, 2019, doi: <http://dx.doi.org/10.1093/ofid/ofz186>.

[75] C. Zheng *et al.*, "Using Natural Language Processing and Machine Learning to Identify Gout Flares From Electronic Clinical Notes," (in English), *Arthritis Care and Research,* vol. 66, no. 11, pp. 1740-1748, 2014, doi: <http://dx.doi.org/10.1002/acr.22324>.

[76] E. Choi, A. Schuetz, W. F. Stewart, and J. Sun, "Using recurrent neural network models for early detection of heart failure onset," *Journal of the American Medical Informatics Association : JAMIA,* vol. 24, no. 2, pp. 361-370, 2017, doi: <https://dx.doi.org/10.1093/jamia/ocw112>.

[77] C.-H. Su, C.-S. Wu, C.-J. Kuo, S.-H. Wang, and H.-J. Dai, "Using text mining to extract depressive symptoms and to validate the diagnosis of major depressive disorder from electronic health records," (in English), *Journal of Affective Disorders,* vol. 260, pp. 617-623, 2020, doi: <http://dx.doi.org/10.1016/j.jad.2019.09.044>.

[78] S. DeLisle *et al.*, "Using the Electronic Medical Record to Identify Community-Acquired Pneumonia: Toward a Replicable Automated Strategy," (in English), *PLoS ONE,* vol. 8, no. 8, p. e70944, 2013, doi: <http://dx.doi.org/10.1371/journal.pone.0070944>.

[79] K. Chaudhary *et al.*, "Utilization of deep learning for subphenotype identification in sepsis-associated acute kidney injury," (in English), *Clinical Journal of the American Society of Nephrology,* vol. 15, no. 11, pp. 1557-1565, 2020, doi: <http://dx.doi.org/10.2215/CJN.09330819>.
