# Supplementary material for: Ensemble machine learning methods in screening electronic health records: A scoping review
Source: Digit Health. 2023 May 9;9:20552076231173225. doi: 10.1177/20552076231173225 (PMC10176785; doi:10.1177/20552076231173225)
Supplement: sj-docx-7-dhj-10.1177_20552076231173225 - Supplemental material for Ensemble machine learning methods in screening electronic health records: A scoping review [file sj-docx-7-dhj-10.1177_20552076231173225.docx]

| **Type of article** | **N (%)** |
| --- | --- |
| All selected articles | 145 |
| Development only | 39 (26.9) |
| Development and validation  🡺Internal validation  🡺External validation  🡺Prospective validation | 105 (72.4)  🡺 79 (54.5)  🡺 20 (13.8)  🡺 6 (4.1) |
| Validation only (external)  🡺External validation | 1 (0.7)  🡺 1 (0.7) |

Supplemental Table 1. Type of studies in the selected articles
