# Supplementary material for: Ensemble machine learning methods in screening electronic health records: A scoping review
Source: Digit Health. 2023 May 9;9:20552076231173225. doi: 10.1177/20552076231173225 (PMC10176785; doi:10.1177/20552076231173225)
Supplement: sj-docx-8-dhj-10.1177_20552076231173225 - Supplemental material for Ensemble machine learning methods in screening electronic health records: A scoping review [file sj-docx-8-dhj-10.1177_20552076231173225.docx]

**Supplemental Table 2. Inclusion Exclusion criteria of diseases and their classification by medical specialties**

| **STATED DISEASE** | **SUGGESTED SPECIALTY** | **INCLUSION** |
| --- | --- | --- |
| acute asthma exacerbation | respiratory medicine | included |
| acute kidney injury | nephrology | included |
| acute organ failure | "other" | excluded symptom not disease |
| alzheimer's disease | neurology | included |
| anaphylaxis | allergy & immunology | included |
| asthma (in children) | respiratory medicine | included |
| atopic dermatitis | dermatology | included |
| atrial fibrillation | cardiology | included |
| bacteremia and fungemia | infectiology | included |
| barrett's oesophagus (mark-be) | gastroenterology | included |
| breast cancer | oncology | included |
| calciphylaxis | "other" | excluded symptom not disease |
| cardiac dysrhythmia | cardiology | included |
| cardiovascular disease | cardiology | included |
| carotid atherosclerosis | neurology | included |
| chronic cough | respiratory medicine | excluded symptom not disease |
| chronic kidney disease | nephrology | included |
| chronic myelogenous leukemia | haematology | included |
| cirrhosis | hepatology | included |
| colorectal cancer | oncology | included |
| congestive heart failure | cardiology | included |
| covid-19 | infectiology | included |
| delirium | mental health | excluded symptom not disease |
| dementia | neurology | included |
| depression | mental health | included |
| depression and anxiety | mental health | included |
| diabetes | endocrinology & metabolism | included |
| diabetes mellitus 2 | endocrinology & metabolism | included |
| diabetic peripheral neuropathy | endocrinology & metabolism | included |
| diabetic retinopathy | ophthalmology | included |
| disseminated intravascular coagulation | haematology | included |
| dysphagia | gastroenterology | excluded symptom not disease |
| endometrial cancer | oncology | included |
| epilepsy | neurology | included |
| familial hypercholesterolaemia | endocrinology & metabolism | included |
| fatty liver disease | hepatology | included |
| fibromyalgia | rheumatology | included |
| gestational diabetes | endocrinology & metabolism | included |
| hand, foot, and mouth disease | infectiology | included |
| heart disease | cardiology | included |
| heart failure | cardiology | included |
| hiv | infectiology | included |
| hypertension | cardiology | included |
| hypertrophic cardiomyopathy | cardiology | included |
| hypoglycaemia | endocrinology & metabolism | excluded symptom not disease |
| influenza | infectiology | included |
| lung cancer | oncology | included |
| macular degeneration | ophthalmology | included |
| major adverse cardiovascular events | cardiology | excluded symptom not disease |
| malignant pleural effusion | oncology | included |
| melanoma | oncology | included |
| mesothelioma | oncology | included |
| myocardial infarction | cardiology | included |
| myopia | ophthalmology | included |
| neonatal sepsis | infectiology | included |
| newborns at risk for autism | mental health | included |
| nonalcoholic steatohepatitis | hepatology | included |
| nontuberculous mycobacterial lung disease | infectiology | included |
| obesity | endocrinology & metabolism | included |
| opioid dependence | mental health | included |
| osteoporosis | rheumatology | included |
| pancreatic cancer | oncology | included |
| postpartum depression | mental health | included |
| post-stroke pneumonia | infectiology | included |
| post-stroke spasticity | neurology | included |
| preeclampsia | obstetrics & gynaecology | included |
| primary hyperparathyroidism | endocrinology & metabolism | included |
| prodromal alzheimer's disease | neurology | included |
| psychosis | mental health | excluded symptom not disease |
| respiratory failure | respiratory medicine | excluded symptom not disease |
| rheumatoid arthritis | rheumatology | included |
| sepsis | infectiology | included |
| sepsis (in children) | infectiology | included |
| sepsis in covid-19 patients | infectiology | included |
| septic shock | infectiology | included |
| stroke | neurology | included |
| suicide (or suicide ideation as they write) | mental health | included |
| systemic lupus erythematosus | rheumatology | included |
| systemic lupus erythematosus births | rheumatology | included |
| systemic sclerosis | rheumatology | included |
| type 2 diabetes | endocrinology & metabolism | included |
| unhealthy drinking | mental health | included |
| urinary tract infections | infectiology | included |
| venous thromboembolism | respiratory medicine | included |
| ventricular arrhythmias | cardiology | included |
| wild-type transthyretin amyloid cardiomyopathy | cardiology | included |
| zika syndrome | infectiology | included |
